# Application of the Discrete Element Method for Manufacturing Process Simulation in the Pharmaceutical Industry

**DOI:** 10.3390/pharmaceutics11080414

**Published:** 2019-08-15

**Authors:** Su Bin Yeom, Eun-Sol Ha, Min-Soo Kim, Seong Hoon Jeong, Sung-Joo Hwang, Du Hyung Choi

**Affiliations:** 1 Department of Pharmaceutical Engineering, Inje University, Gyeongnam 621-749, Korea; 2 College of Pharmacy, Pusan National University, Busandaehak-ro 63 beon-gil, Geumjeong-gu, Busan 46241, Korea; 3 College of Pharmacy, Dongguk University, Goyang 410-820, Korea; 4 College of Pharmacy, Yonsei University, 85 Songdogwahak-ro, Yeonsu-gu, Incheon 21983, Korea

**Keywords:** discrete element method, manufacturing process simulation, contact model, input parameter, calibration method

## Abstract

Process simulation using mathematical modeling tools is becoming more common in the pharmaceutical industry. A mechanistic model is a mathematical modeling tool that can enhance process understanding, reduce experimentation cost and improve product quality. A commonly used mechanistic modeling approach for powder is the discrete element method (DEM). Most pharmaceutical materials have powder or granular material. Therefore, DEM might be widely applied in the pharmaceutical industry. This review focused on the basic elements of DEM and its implementations in pharmaceutical manufacturing simulation. Contact models and input parameters are essential elements in DEM simulation. Contact models computed contact forces acting on the particle-particle and particle-geometry interactions. Input parameters were divided into two types—material properties and interaction parameters. Various calibration methods were presented to define the interaction parameters of pharmaceutical materials. Several applications of DEM simulation in pharmaceutical manufacturing processes, such as milling, blending, granulation and coating, were categorized and summarized. Based on this review, DEM simulation might provide a systematic process understanding and process control to ensure the quality of a drug product.

## 1. Introduction

Currently, the pharmaceutical industry faces a number of regulatory and economic challenges that are related to process development [1]. From a regulatory perspective, the development of the manufacturing process should be based on systematic process understanding and process control to ensure the quality of the drug product in accordance with the quality by design (QbD) approach defined by an international conference on harmonization guidelines (ICH Q8: pharmaceutical development) [2]. Therefore, it is important to obtain adequate information related to the pharmaceutical process to establish the correlation between the quality of the drug product and the process parameters [1]. In addition, the development of the manufacturing process may require considerable time and resources from an economic perspective. These challenges may result from the lack of cost-effective and reliable modeling tools of unit operation development in the pharmaceutical industry, in contrast to other chemical industries [3]. Therefore, it is necessary to apply the modeling tools to the process, not only to overcome the challenges of regulatory and economic aspects but also to develop a more efficient and robust process [1]. In response to this necessity, the modeling of the manufacturing process has been become increasingly important, as it can be applied to equipment design, improving process efficiency, scale-up and unit operation development in the pharmaceutical industry [4]. 

In general, the modeling tools for the manufacturing process are divided into three types, empirical models, mechanistic models and hybrid models. The empirical models include multivariate analysis (MVA), artificial neural network (ANN) and design of experiments (DoE), based on empirical, semiempirical or statistical methods. The models collect large amounts of data from experiments and generate models based on these data. Therefore, the empirical models have good predictability in the inside area of experimental space but these are limited in their predictability outside the area of experimental space. The mechanistic models include in silico tools, such as computational fluid dynamics (CFD), finite element method (FEM) and discrete element method (DEM). These mechanistic models generally perform the process simulation [5]; they capture the underlying physical phenomena through fundamental first principles such as mass, momentum and energy. Therefore, the mechanistic models have good predictability, not only within the experimental space but also outside this area. In addition, such models are preferred as they provide scientific insight into the unit operation in the pharmaceutical industry [6,7,8,9]. The hybrid models are a combination of the mechanistic model and empirical model; they include scale-up equations and property estimation. The models can describe process knowledge in the mechanistic model on the basis of first principles and any gaps in process knowledge can be addressed by using an empirical model based on experimental data [8]. 

The mechanistic models have been applied widely for the prediction of the effect of process parameters and present insights into unit operations in the pharmaceutical industry, following dramatic advances in computer engineering. CFD—a popular and powerful modeling tool that provides insights into mathematical physics and numerical methods for fluid flow [10]—numerically solves mass, momentum and energy balance with Euler or Navier-Stokes equations [4]. The model can be applied to the simulation of pharmaceutical processes, such as granulation, where fluid motion acts, as it is suitable for systems consisting of fluids [11]. Another mechanistic model, FEM, is a numerical approach similar to CFD and divides the process into finite elements or volumes. FEM can be classified as the Eulerian or Lagrangian model, depending on how the finite element is defined [12]. This method can predict the dynamics of powders by using partial differential equations that describe mass, momentum and energy balance [6]. The FEM is suitable for application in process simulation for particles considered elastic or elastoplastic in systems composed of dense solids, such as the compaction process [4,13,14]. 

Among the mechanistic models, one of the more commonly used DEM is a Lagrangian model that simulates the velocity, position and motion of individual particles [6,9]. DEM can provide information, such as trajectory or forces acting on individual particles, which is difficult to obtain experimentally; moreover, it can address the size distribution of individual particles that cannot be handled in a Eulerian model [15]. In addition, DEM has the strength of allowing the parametric study to examine the effects of individual particles, process conditions or equipment design on the quality of the drug product [4]. Therefore, DEM can be applied to various simulations of unit operations such as blending, granulation and coating [16,17,18]. Moreover, DEM in combination with CFD or FEM can be applied to the processes simulation related to the granulation and compaction [19,20,21,22]. Despite the various advantages of manufacturing process simulation using DEM, there is a limit to the computational burden, which increases as the number of particles increases in the simulation. However, this limitation has been overcome somewhat with the growth of computer hardware and the advent of commercially available software that uses parallel computing techniques [4,5]. In addition to these computational advances, DEM has recently attracted attention as a valuable model that provides insight into the process in the pharmaceutical industry [6]. 

This review focused on the application of DEM simulation in the pharmaceutical industry. The basic elements of DEM and its implementations in pharmaceutical processes are comprehensively presented. A theory of DEM has been described with hard-sphere and soft-sphere models. To calculate the contact force acting on the particle-particle and particle-geometry interactions, contact models are categorized as the particle properties involved in the pharmaceutical process. In addition, the non-contact forces such as van der Waals force, liquid bridge force and electrostatic force is described. DEM applications in the most frequently used manufacturing processes in the pharmaceutical industry, such as milling, blending, granulation and coating, are comprehensively summarized. 

## 2. Comprehensive Theory of Discrete Element Method 

DEM is a numerical method for predicting mechanical dynamics, such as position, velocity and motion of individual particles introduced by Cundall et al. [23]. The underlying principles of DEM are as follows: (a) the force exerted by neighboring particles or boundaries for each particle is calculated in a single time step using the contact model; (b) Newton’s second law is applied to calculate the particle velocity; (c) based on the same principle, the rotational momentum balances are solved to track the rotational velocity of particles; (d) the new position of the particle is calculated for a time-step length. This procedure is applied to each particle in a single time step and repeated for each time step [17,24,25,26]. 

In general, DEM is classified into four models—cellular automata (CA), Monte Carlo method, hard-sphere model and soft-sphere model [9]. CA constrains the particles to a lattice and sets the particle motion by using simple rules obtained through experimental results. CA is a computationally simple and efficient model for the investigation of physical phenomena and allows qualitative prediction of flow dynamics. CA has been applied to investigate the flow dynamics in rotating cylinders and inclined chutes [27,28], as well as to evaluate blending and segregation, including the effects of shaking and percolation [29,30]. However, CA is currently limited for the application of the quantitative study of particles, as its validity for quantitative prediction has not been proven [4,9]. Monte Carlo method models the motion of particles associated with realistic equations of motion through the calculation of random arrangements of particles in the system [31]. The Monte Carlo method is a computationally efficient model as CA and has been applied to the modeling of granular materials. Several studies have been conducted to investigate the dynamic of granular materials, such as hopper flow, blending and segregation, by using the Monte Carlo method [32,33,34]. However, in the pharmaceutical industry, the Monte Carlo method has not been as common as the hard-sphere and soft-sphere models. The models can predict quantitative results that are in good agreement with actual experimental results, as well as they are more flexible in application. Therefore, the models might be preferred in the pharmaceutical industry [4,9,15]. The advantages and disadvantages of the hard-sphere and soft-sphere models have been explained in detail in various studies [4,5,35]. In addition, the comprehensive theory of these two models is described in the following sections. 

### 2.1. Hard-Sphere Model

**Figure 1:**
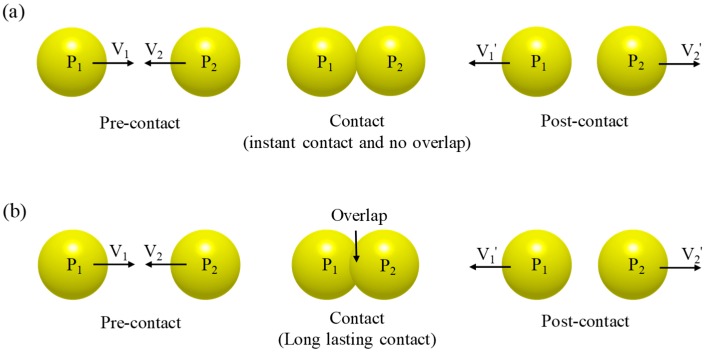
Simple schematic diagram of (**a**) hard-sphere model and (**b**) soft-sphere model. (V_1_ and V_2_: the velocity of each particle before contact; V_1_′ and V_2_′: the velocity of each particle after contact).

In the hard-sphere model, the particles are considered to be rigid and the particle contact, which is determined by the binary contact rule, is assumed to be instantaneous, as shown in Figure 1a [36]. This assumption suggests that the hard-sphere model is suitable for highly agitated or gravity-free conditions [9]. The hard-sphere model can be referred to as an event-driven approach, as it increases the simulation time by processing one by one according to the order in which the particle contact occurs. Therefore, the hard-sphere model might be computationally efficient when applied to a not too dense system [37,38]. However, this model is limited for applications where multiple contacts occur at the same instant, as the computational burden increases dramatically due to the update of information such particle velocity and trajectory whenever particle contact occurs [5]. To alleviate the computational burden of the hard-sphere model, a hybrid model was proposed by Hopkins [39]. This model, which is referred to as the hard-sphere/overlap technique, combines the hard-sphere model with an overlap strategy. In the hybrid model, the hard-sphere model is incorporated with the equation of particle motion between contacts at the specified time step. Therefore, it is referred to as a time-driven approach and can be applied to a system with a relatively large number of particles [4]. The hard-sphere model has been applied to a wide range of studies to address the granular flow in the manufacturing process, as it was first applied to the study that describes the shear-flow of granular material in a two-dimensional simulation [38,40]. In addition, the hard-sphere model has been applied to describe the segregation of granular material in the fluidized bed, as well as to investigate the granular flow in the inclined plane or chutes [41,42]. 

### 2.2. Soft-Sphere Model

The soft-sphere model may be the most common and flexible in DEM [9]. In the soft-sphere model, it is assumed that the particle contact is lasting, as shown in Figure 1b and that multiple contacts between particles are possible, as well as binary contact [36]. These assumptions for particle contact indicates that the soft-sphere model is desirable for the investigation of long-lasting and multiple particle contact in a high dense system [4,5,37]. The soft-sphere model is referred to as a time-driven approach, as it sets a time step in which the contact force is calculated. Therefore, the time step should be carefully set for accurate simulation. The smaller time steps allow accurate integration of the resulting particle equations but can dramatically increase the computational time in the simulation [4,38]. 

In the soft-sphere model, the simulation process is relatively simple and has the following steps: (a) Setting the particles and equipment properties into the computational domain; (b) Inserting the particles into the computational domain by defining the position and velocity; (c) Detecting the particle-particle and particle-geometry contacts; (d) Calculating the force acting on each particle by using an appropriate contact model; (e) Determining the particle acceleration by using Newton’s second law, which is integrated in time to determine the new particle states such as position and velocity. This process is repeated to track the dynamics of particles over a long period of time until the desired result is obtained [4,9]. The soft-sphere model has been applied to the various manufacturing process, such as for processing the granular materials in the pharmaceutical industry owing to its suitability in highly dense systems related to the particle concentrations [39]. A number of studies have been performed using the soft-sphere model to simulate the dynamics of granular materials in blending, high shear granulation, coating, milling and die filling processes [17,26,43,44,45,46,47,48].

### 2.3. Contact Model

**Figure 2:**
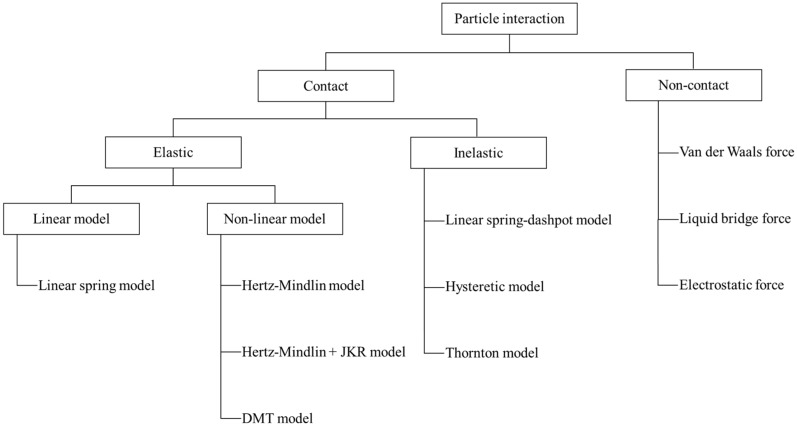
Classification of particle interaction force models by contact force and non-contact force.

Modeling the contact between particles might be the most important role in DEM simulation. This is performed by a contact model that calculates the contact forces acting on the particle-particle and particle-geometry interactions [4]. The contact model can be categorized depending on the physical properties of particles such as elasticity, plasticity, viscosity, dry friction and adhesion [49]. In this review, regarding contact models, we focused on the elastic and inelastic properties of particles (Figure 2), which are commonly used in pharmaceutical manufacturing process simulations. Detailed descriptions of the contact models are presented in the following sections.

**Figure 3:**
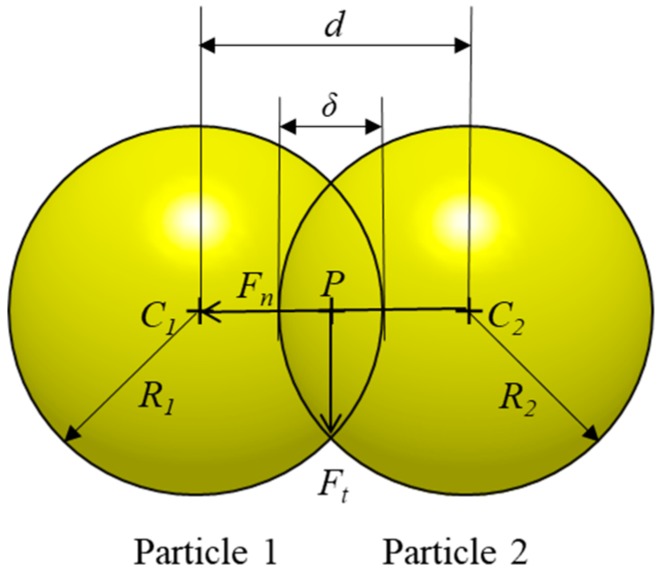
Illustration of normal and tangential forces involved in the contact between particles. (*C*_1_ and *C*_2_: the center of particle 1 and particle 2; *R*_1_ and *R*_2_: the radius of particle 1 and particle 2; d: distance between the *C*_1_ and *C*_2_; *δ*: the overlap between particle 1 and particle 2; *P*: the center point of overlap; *F_n_* and *F_t_*: the contact force in the normal direction and the contact force in the tangential direction, respectively.).

Generally, contact between two particles occurs in the finite area due to the deformation of the particle and the contact traction distribution in this area consists of the normal and tangential plane [15]. Therefore, as shown in Figure 3, the contact force acting on particles is divided into two types of components—normal and tangential [49]. The overlap (*δ*) between the two contacting particles can be calculated from the equation *δ = R*_1_ *+ R*_2_ − *d. R*_1_ and *R*_2_ are the radius of particle 1 and 2, respectively and *d* is the distance between the centers of particle 1 and 2 (*C*_1_ and *C*_2_). The $\vec{C_{1}C_{2}}$ vector covers the normal contact direction and the line perpendicular to $\vec{C_{1}C_{2}}$ covers the tangential contact direction [50]. Besides these normal and tangential contact forces, various forces act on the particles such as damping forces that dissipate after contact between two particles, friction forces and cohesive forces. These forces involved between the particles are defined based on the contact model, which is then used to determine the acceleration of particles. Subsequently, the calculated acceleration is integrated in time and used to update the new state of the particle (e.g., position and velocity) [4]. 

#### 2.3.1. Elastic Contact Model

##### Linear Spring Model

**Figure 4:**
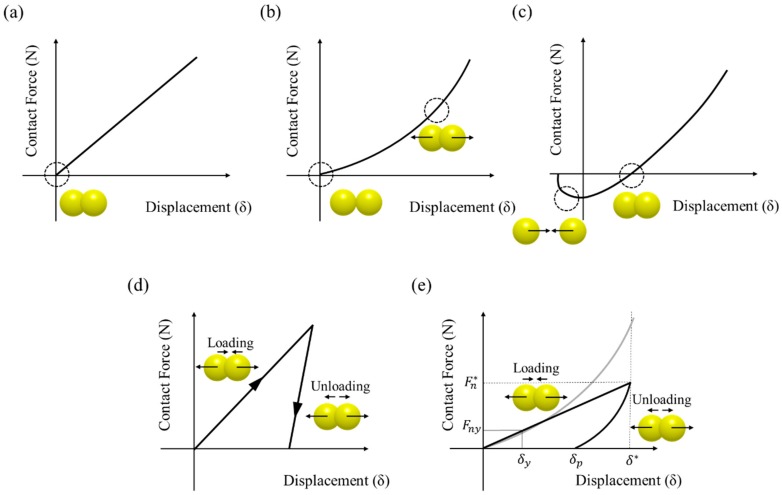
Relationship between force and displacement in various contact models: (**a**) linear spring model, (**b**) Hertz-Mindlin model, (**c**) Hertz-Mindlin + JKR model, (**d**) hysteretic model and (**e**) Thornton model.

In general, elastic contact models are classified as either linear elastic or nonlinear elastic models. The linear elastic model is simplified as a spring but the nonlinear elastic model is based on the more complex Hertz theory [51,52,53]. The linear spring model is the basic contact model that represents the linear relationship between force and displacement, as shown in Figure 4a. The displacement increases as the contact force increase. This linear relationship is derived from the following equations [51]:

(1) $$F_{n}=-K_{n}\delta_{n}$$

(2) $$F_{t}=-K_{t}\delta_{t}$$

where *F_n_* and *F_t_* denote the contact forces in the normal and tangential direction, respectively and *δ_n_* and *δ_t_* are the normal and tangential displacements, respectively. *K_n_* and *K_t_* are the spring constant of the particles involved in the contact in the normal and tangential direction, respectively. The linear spring model considers that two particles in contact are both normally and tangentially connected by linear spring [54]. Therefore, if the contact between particles is modeled by a linear spring model based on Equations (1) and (2), then energy is not consumed and the contact is considered completely elastic. In most practical cases, however, some kinetic energy is dissipated by plastic deformation and/or some kinetic energy is converted to another energy. Therefore, a linear spring model has a limitation in application to particle contact modeling [55]. 

##### Hertz-Mindlin Model

The Hertz-Mindlin model is a representative nonlinear elastic model that describes the nonlinear relationship between normal force and displacement [53]. The nonlinear relationship between force and displacement for the Hertz-Mindlin model is shown in Figure 4b. In the Hertz-Mindlin model, the contact between two particles in the normal direction was proposed based on Hertz theory [56], whereas the contact between two particles in the tangential direction was proposed by Mindlin and Deresiewicz theory [57]. However, the complete Hertz-Mindlin model described in previous studies has a computational limitation due to its considerable complexity [53,58,59,60]. Moreover, the complete Hertz-Mindlin model is computationally time-consuming when it involved in the contact of a large number of particles [15]. Therefore, the Hertz-Mindlin model was simplified to the Hertz-Mindlin no slip model based on Hertz’s theory in the normal direction and Mindlin’s no slip improved model in the tangential direction. In the Hertz-Mindlin no slip model, the normal and tangential contact forces are calculated from the following equations, respectively [58]:

(3) $$F_{n}=-\frac{4}{3}E_{eq}\sqrt{R_{eq}}\cdot \delta_{n}^{3/2}$$

(4) $$F_{t}=8G_{eq}\sqrt{R_{eq}\cdot \delta_{n}}\cdot \delta_{t}$$

where *E_eq_*, *R_eq_* and *G_eq_* are the equivalent Young’s modulus, equivalent radius and equivalent shear modulus for the two particles (particle 1 and particle 2) involved in the contact, respectively. These parameters are described in detail in the previous study [61]. The Hertz-Mindlin no slip model has been applied to the many manufacturing process simulation due to its accuracy of the result prediction in the pharmaceutical industry. Several studies have been reported on applying the Hertz-Mindlin no slip model to various manufacturing processes such as milling, blending, granulation and coating [61,62,63,64]. 

##### Hertz-Mindlin + JKR Model and DMT Model

As another nonlinear elastic model, several models have been developed to model the contact of cohesive particles. The Hertz-Mindlin + JKR model was proposed by Johnson, Kendall and Roberts based on Hertz theory [65]. This model describes the adhesive theory using a balance between stored elastic energy and loss of surface energy. The nonlinear relationship between force and displacement for the Hertz-Mindlin + JKR model was suggested in Figure 4c. The Hertz-Mindlin + JKR model has the opposite force owing to the pulling force that results from the adhesion effect of the particles when the contact between particles is initiated. The Hertz-Mindlin + JKR model also predicts larger contact areas than the contact areas based on conventional Hertz theory due to the cohesion [49]. In the Hertz-Mindlin + JKR model, the contact force is derived from the following equation [66]:

(5) $$F_{JKR}=\frac{4E_{eq}a^{3}}{3R_{eq}}-\sqrt{8\pi a^{3}\Delta \gamma E_{eq}}$$

where *a* is the contact area and *γ* is the surface energy. The Hertz-Mindlin + JKR model has been applied to several manufacturing processes involving cohesive particles in the pharmaceutical industry. Deng et al., studied the blending process of cohesive particles by applying the Hertz-Mindlin + JKR model to account for the cohesive force between particles in DEM simulation [67]. In addition, Behjani et al. studied the wet granulation process based on DEM simulation with the Hertz-Mindlin + JKR model to investigate the formation of granules [68]. 

Additionally, a DMT model was proposed by Derjaguin, Muller and Toporov based on the cohesion at the contact periphery in contrast with the Hertz-Mindlin + JKR model based on the surface energy [69]. The contact force in the DMT model can be calculated from the following equation [66]:

(6) $$F_{DMT}=\frac{4E_{eq}a^{3}}{3R_{eq}}-2\pi R\Delta \gamma$$

Therefore, the DMT model is suitable for hard materials, such as solids with a small tip radius and low surface energy, whereas the Hertz-Mindlin + JKR model is desirable for soft elastic material with a large tip radius and high surface energy [66]. 

#### 2.3.2. Inelastic Contact Model

##### Linear Spring-Dashpot Model

Unlike the previous elastic contact model that focused on the accumulation of energy, the inelastic contact model was suggested to model the dissipation of energy when plastic deformation between particles occurs [60]. The most common and intuitive model for inelastic contact models is the linear spring-dashpot (LSD) model proposed by Walton based on the dashpot model used by Cundall and Strack [23,70]. In the LSD model, normal contact force is calculated using the equation [71]:

(7) $$F_{n}=-K_{n}\delta_{n}+\eta_{n}V_{n}$$

Equation (10) is composed of linear spring and dashpot components, where a linear spring describes the repulsive forces and dashpot dissipates some of the relative kinetic energy [72]. The $\eta_{n}v_{n}$ corresponding to the dashpot component denotes normal damping force, which is expressed by multiplying the normal damping coefficient ($\eta_{n}$) and relative velocity of the contacting particles in the normal direction $\left(V_{n}\right)$ [55]. The tangential contact model is calculated by applying the linear spring limited by Coulomb’s law of friction (i.e., $\mu F_{n}$; μ is the coefficient of friction) using the following equation [71,72,73]:

(8) $$F_{t}=\min\left\{\mu F_{n},\;K_{t}\int V_{t}d_{t}+\eta_{t}V_{t}\right\}$$

where the integral term, which corresponds to the linear spring component, means the incremental spring that stores energy resulting from the tangential motion and models the elastic deformation due to contact in the tangential direction. $\eta_{t}V_{t}$, which is the dashpot component, denotes tangential damping force, as defined by multiplying the tangential damping coefficient ($\eta_{t}$) and relative velocity of the contacting particles in the tangential direction ($V_{t}$) [72]. The LSD model is widely used for process simulation in the pharmaceutical industry owing to its simplicity and robustness [74]. Several studies using the LSD model in various pharmaceutical manufacturing processes, such as blending, coating and high shear wet granulation, have been reported [17,26,44]. 

##### Hysteretic Model

The basic concept of the inelastic model is that it uses various spring constant at the loading, unloading and reloading stages [53]. Accordingly, Walton and Braun developed the linear contact model, referred to as the hysteretic model, which considered plastic deformation [75]. The relationship between force and displacement for the hysteretic model was as suggested in Figure 4d. In this model, a partially latched spring force-displacement model is applied in the normal direction and the approximation of Mindlin and Deresiwicz theory is used for the case of the constant normal force in the tangential direction [15,74]. Therefore, this model is limited as it describes the plastic deformation only in the normal direction [60]. The contact force in the normal direction according to the loading and unloading stage of spring constant is calculated from the following equation [15,53]:

(9) $$F_{n}=\{\begin{array}{c} -K_{1}\delta_{n},\;{\dot{\delta }}_{n}>0\\ \left(loading\right)\\ -K_{2}\left(\delta_{n}-\delta_{n0}\right),\;{\dot{\delta }}_{n}<0\\ \left(unlodaing\right) \end{array}$$

where *K*_1_ and *K*_2_ are the spring constants in the loading and unloading stages, respectively. $\delta_{n0}$ denotes the normal displacement when the unloading curve goes to zero (Figure 4d). This hysteretic model has been used in many manufacturing process simulations involving inelastic particles in the pharmaceutical industry. Chudhuri et al. studied the effect of cohesion in the blending and size segregation of binary mixture in blending process simulation with the hysteretic model [76]. In addition, Sahni et al. used the hysteretic model in coating process simulation to obtain the optimal blending condition in a pan coater [77]. 

##### Thornton Model

Another simplified model explaining plastic deformation was developed based on Thornton’s theory [78]. This simplified model has been proposed for normal contact between two elastic, perfectly spherical plastic particles. The Thornton model is based on the Hertz theory for normal force-displacement relationship during the initial elastic loading but it suggests that plastic deformation occurs if the limiting contact pressure is reached at the center of the contact area, as shown in Figure 4e. The limiting contact pressure ($P_{y}$) is defined as the following equation [60,74]:

(10) $$P_{y}=\frac{3F_{ny}}{2\pi \delta_{y}^{2}}$$

where $F_{ny}$ and $\delta_{y}$ denote the normal contact force and displacement, respectively, when the pressure at the center of contact area reaches the $P_{y}$. In other words, the Thornton model proposes that the normal contact force during the initial elastic loading ($\delta <\delta_{y}$) is calculated as Equation (3) but the normal contact force during plastic loading ($\delta >\delta_{y}$) is defined as following equation [74]:

(11) $$F_{n}=F_{ny}+\pi R_{eq}P_{y}\left(\delta -\delta_{y}\right)$$

In addition, the normal contact force during elastic unloading is calculated based on the Hertz theory but with a radius curvature of contact area (referred to as $1/R_{eqp}$), corresponding to the point of maximum normal contact force ($\delta_{p}$) [74,78,79]:

(12) $$F_{n}=\frac{4}{3}E_{eq}\sqrt{R_{eqp}}{\left(\delta -\delta_{p}\right)}^{3/2}$$

(13) $$R_{eqp}=\frac{4E_{eq}}{3F_{n}^{*}}{\left(\frac{2F_{n}^{*}+F_{ny}}{2\pi P_{y}}\right)}^{3/2}$$

(14) $$\delta_{p}=\delta^{*}-{\left(\frac{3F_{n}^{*}}{4E_{eq}\sqrt{R_{eqp}}}\right)}^{2/3}$$

where $F_{n}^{*}$ denotes the maximum normal contact force from which unloading commenced and $\delta^{*}$ means the maximum relative displacement at the point of unloading (Figure 4e).

### 2.4. Non-Contact Force

Besides the contact forces, non-contact forces such as van der Waals force, liquid bridge force and electrostatic force can act simultaneously or successively between the particles to affect their micro- or macroscopic dynamics in the manufacturing process [15,80]. In addition, the effect of these non-contact forces may be more significant in the pharmaceutical process involving fine particles or liquid [4]. Van der Waals force denotes the attractive intermolecular forces that composed of various type of interaction such as dipole-dipole, dipole-non-polar and non-polar-non-polar [81]. Van der Waals force can be calculated using the two types of approaches such as the Hamaker-based theory approach and the Lifshitz-based theory approach [82]. The former is based on the sum of the individual interactions between molecules [83] and the latter is based on the quantum field theory and considers the interacting molecules as continuous macroscopic materials [84]. In DEM simulation, van der Waals force ($F_{vdW}$) is commonly calculated based on the Hamaker-based theory approach, which is defined by the following equation [15,85]:

(15) $$F_{vdW}=\frac{HR}{6h^{2}}$$

where *h* is the separation distance between spherical particles; *H* is the Hamaker constant; *R* is the radius of the spherical particle. The Equation (15) indicates that van der Waals force becomes infinite when the spherical particle contact (*h* = 0), which can be caused a significant problem in DEM simulation. Therefore, the minimum separation distance referred to as cut-off distance is taken into account in the calculation of van der Waals force [15]. There is no general value of cut-off distance, it is determined to within the range of 0.1–1.0 nm in several studies related to DEM simulation [82,86,87,88]. 

The liquid bridge force is the cohesive force generated by the formation of the liquid bridge between two wet particles [89]. In general, the liquid bridge force ($F_{lb}$) is defined by the following equation as the sum of the surface tension and the force due to the reduced hydrostatic pressure in the liquid bridge [90,91]:

(16) $$F_{lb}=2\pi \gamma^{\prime }R\sin\emptyset \sin\left(\emptyset +\theta \right)+\pi R^{2}\Delta P\sin^{2}\emptyset$$

where $\gamma^{\prime }$ is the liquid surface tension; ∅ is the half-filling angle; θ is the contact angle; ΔP, which is given by Laplace-Young equation, is the reduced hydrostatic pressure within the liquid bridge. The descriptions of these parameters have been covered in detail in previous studies [15,91,92]. However, it is complicated to calculate the liquid bridge force in that it is difficult to obtain the information on wetting of particles and the liquid distribution around the particles [93]. Therefore, some simplified solutions have been proposed to easily calculate the capillary force in DEM simulation [94,95,96,97].

The electrostatic force is the force acting on the charged particles. Generally, the electrostatic force ($F_{e}$) between particles is determined based on the Coulomb’s law, which can be expressed by the following equation as a function of the charge of particles and the distance between particles [98,99]:

(17) $$F_{e}=\frac{1}{4\pi \varepsilon_{0}}\frac{q_{1}q_{2}}{d^{2}}\hat{d}$$

where $\varepsilon_{0}$ is the permittivity of free space; *d* is the distance between the particle’s center; $q_{1}$ and $q_{2}$ are the charges of particle 1 and particle 2, respectively. The explanations of these parameters have been discussed in detail in previous studies [100,101]. 

Several DEM simulation using non-contact forces were studied for the pharmaceutical manufacturing process. Yang et al. studied DEM simulation to confirm the effect of van der Waals force and liquid bridge force in the packing behavior of cohesive particles [102]. Rhodes et al. investigated the influence of van der Waals force and liquid bridge force on fluidization characteristics using DEM simulation [103]. In addition, Peng et al. studied the aggregation in suspensions using DEM simulation taking into account van der Waals force and electrostatic force [104]. 

### 2.5. Considerations of Computational Time for DEM Simulation

There are limitations in terms of computational time in the DEM simulations applied in pharmaceutical manufacturing processes. This problem can be worsened in the pharmaceutical industry, where the information of particles involved in the manufacturing process is further complexed. Generally, computational time in DEM simulation is expressed by a combination of several factors as follows—time step ($\Delta T_{step}$), number of particles, particle shape, particle size and shear modulus. Time step, which is the time between each iteration, is usually set to be lower than the critical time step defined by the fraction of natural frequency of the equivalent mass-spring system. The critical time step (referred to as Rayleigh time step) is calculated as follows [105]:

(18) $$\Delta T_{step}<\Delta T_{critical}=T_{Rayleigh}=\frac{\pi R\sqrt{\frac{\rho }{G}}}{0.1631v+0.8766}$$

where the v, R, G and R denote the Poisson’s ratio, particle radius, shear modulus and particle density, respectively. In other words, time step is primarily influenced by particle size, particle density and shear modulus. Based on Equation (18), various approaches have been developed to reduce computational burden in DEM simulation. One of these approaches is to reduce computational time by scaling up the particle size to decrease the number of particles in the manufacturing process [106,107]. Another approach is to reduce shear modulus to such an extent that it does not affect the overall dynamic of the particles to achieve a reasonable computational time [108,109]. Detailed descriptions of these approaches are presented in the following section, in which the input parameters in DEM simulation are described. Besides adjusting the properties of particles, the use of high-performance clusters and parallel computing technology can significantly alleviate the computational time limitations [105,110]. 

### 2.6. Input Parameters for DEM Simulation

To accurately simulate the particle dynamics by using DEM, the input parameters related to particle contact should be clearly defined [111,112]. In general, the input parameters are divided into two types, material properties and interaction parameters. The former includes density, particle shape, particle size, shear modulus and Poisson’s ratio and the latter includes the coefficient of restitution, coefficient of static friction and coefficient of rolling friction. In addition, the interaction parameters may include additional parameters, such as plastic or viscous damping and adhesion coefficients dependent on the applied contact model [49]. 

#### 2.6.1. Material Properties

##### Particle Shape

**Figure 5:**
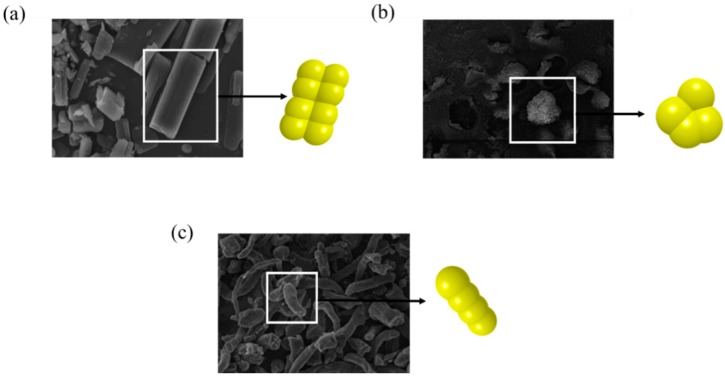
Description and scanning electron microscopy (SEM) images of pharmaceutical particle shapes with the glued-sphere approach: (**a**) amlodipine, (**b**) celecoxib and (**c**) croscarmellose sodium.

The shape of particles is one of the important input parameters to be considered in DEM simulation. Thus, the particle shape should be clearly defined for the accurate prediction of particle dynamics. In general, spherical particles are preferred in DEM simulation as contact is easily detected and the orientation of the particles does not to be determined [112]. But, materials such as particles, granules and tablets used in manufacturing processes in the pharmaceutical industry are not restricted to spheres. Therefore, several irregular shapes, such as polygons, ellipsoids, superquadrics and sphero-cylinders, have been proposed to define the shape of non-spherical particles. In particular, the definition of non-spherical shape using superquadrics shape has attracted attention in that it can address approximately 80% of all particle shapes, as well as the high reproducibility of accurate particle shape [26,113,114]. Therefore, the possibility of superquadrics shape to define the shape of particles and tablets related to the pharmaceutical process simulation has been extensively studied. Cleary et al. performed a 3-dimensional simulation study on the particles flow in the hopper using the superquadrics shape [71] and Cleary investigated the blending performance of plough share blender with realistic shaped particle described by superquadrics shape [115]. In addition, Delaney et al. conducted the milling simulation study with superquadrics shape to provide insight into the breakage mechanisms [116]. However, the definition of non-spherical shapes using such irregular shapes is quite computationally inefficient due to complicated algorithms for contact detection as compared to spheres [117,118,119]. To alleviate this computational inefficiency and define the non-spherical shape, the glued-sphere approach (referred to as the clumped sphere approach) that connects or overlaps multiple spheres has been proposed to define various particle shapes, as shown in Figure 5.

**Figure 6:**
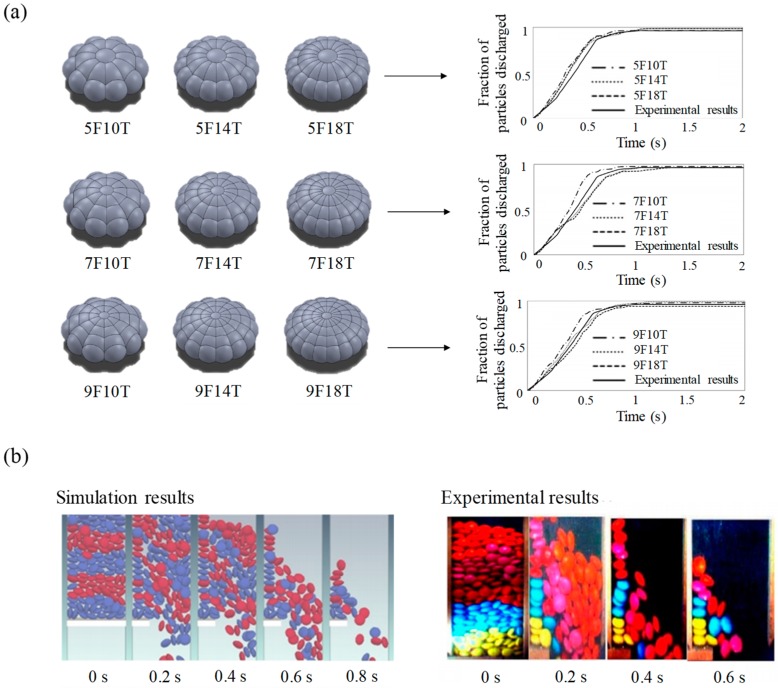
Application of a glued-sphere approach: (**a**) particle shape defined by glued-sphere approach and comparison of fraction of particles discharged in the hopper with actual particles and (**b**) comparison of flow dynamics between simulation results and experimental results [120]. The figures were slightly modified with permission from Elsevier, 2018.

This approach has been commonly applied in simulations involving various material shapes owing to its simplicity of spherical contact detection. Khazeni et al. used the glued-sphere approach to simulate the hopper discharge of elliptical particles [120]. In this study, a total of nine particle shapes were defined by the glued-sphere approach, as shown in Figure 6a. The naming method for each sphere is XFYT; X denotes the number of spheres seen from the front view and Y means the number of repeated radial patterns for the particle center seen from the top view. The fraction of the particles discharged from simulations and experiments were obtained from an orifice of 70 mm and they showed good consistency. Moreover, similar results were obtained by comparing the images of the particle dynamics in the simulation and experiment in the 70 mm orifice, as shown in Figure 6b. Based on these results, it can be inferred that a similar result with the experimental result is obtained by using the glued-sphere approach in the simulation. Besides this study, several studies have been performed by using the glued-sphere approach for an accurate description of complex tablet shapes [18,26,121]. The glued-sphere approach was also applied to define non-spherical particulate materials (e.g., granules and pellets) [105,122]. However, the glued-sphere approach has limitations related to the inaccurate tendency of particle contacts and the computational burden in simulations because this approach uses multiple spheres to define particle shapes [117,123,124]. To overcome these limitations, it is preferred to use a moderate number of spheres that do not require long computational time but can define the described particle shape well [18]. 

In addition, the non-spherical particle shape can be described using GPU-based DEM software, which is being commercialized recently. Based on the use of GPU-based DEM software, the contact detection can be resolved sufficiently simple and fast, though not as much as spheres [125,126]. Govender et al. investigated the hopper discharge of irregular non-convex particle shapes using GPU-based DEM software [127]. Also, Govender et al. studied the effect of particle shape (i.e., spheres, cubes, scaled hexagonal prism, bilunabirotunda, truncated tetrahedral and mixed particle system) on blending uniformity [128]. In this study, each particle shape was defined by GPU-based DEM software. These studies have demonstrated the computational efficiency of GPU-based DEM software for processing millions of non-spherical particles. Therefore, GPU-based DEM software can be widely applied in the pharmaceutical industry involving a number of non-spherical particle shapes to accomplish the process simulation with a reasonable computational time. 

##### Particle Size

**Figure 7:**
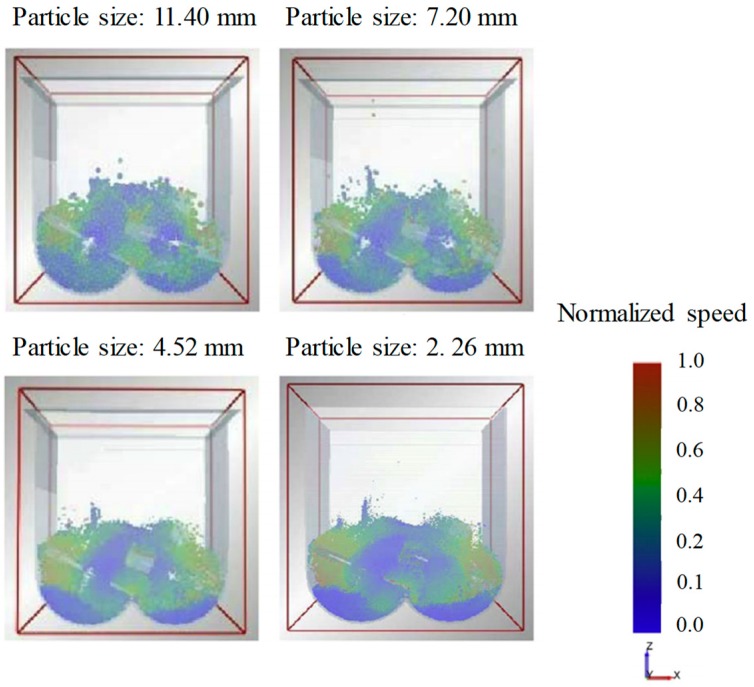
Change in flow pattern as particle size increases in paddle blender [130]. The figure was slightly modified with permission from Elsevier, 2011.

The particle size should also be clearly defined to predict accurate particle dynamics. In general, the particle size used in the pharmaceutical manufacturing process is mostly in the micro and submicron range. However, significant computational time is required to simulate the dynamics of particles within these size ranges. To overcome this computational burden, the particle size can be scaled-up, as long as it does not affect the overall particle dynamics [17,44,129]. Therefore, DEM-based simulations of the pharmaceutical manufacturing process are commonly performed by applying scaled-up particle sizes. Hassanpour et al. conducted a blending simulation to investigate the flow patterns of particles at different sizes in a paddle mixer [130]. In this study, the same particles ranged in size from 2.26, 4.52, 7.20, to 11.40 mm; and the number of particles corresponding to each size to have a 100% fill level in the mixer was 500,000, 60,000, 15,000 and 7000, respectively. In addition, the real time required to simulate 10 s for each size using a desktop quad core Intel^®^ processor was 580, 54, 6 and 3 h, respectively. The flow pattern of the particles at each size obtained at the identical time after the blending simulation is shown in Figure 7. The flow patterns are qualitatively consistent with each other in all blending simulation, despite the different particle sizes. Therefore, it can be concluded that the scaling-up of particle size is efficient to an extent that it does not affect the overall particle dynamics to perform the simulation within a reasonable time.

**Figure 8:**
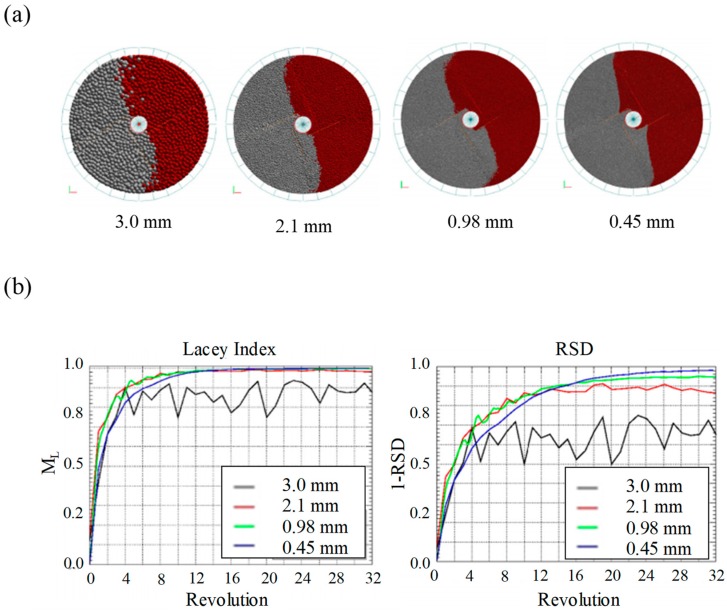
Blending simulation using scaled-up particles: (**a**) range of particle size; (**b**) the Lacey index (*M_L_*) and relative standard deviation (RSD) for each particle size [110]. The figures were slightly modified with permission from Elsevier, 2010.

In addition, Radeke et al. performed a blending simulation with scaled-up particles to reduce computational time [110]. The particle size ranged from 0.45, 0.98, 2.1, to 3.0 mm, as shown in Figure 8a; the number of particles of each size was 7,680,000, 786,000, 76,800 and 7680, respectively; and the simulation time of each size was 1355, 806, 545 and 350 s, respectively. Particles corresponding to each size were applied in the blending simulation and relative standard deviation (RSD) and Lacey index were evaluated to reflect the blending homogeneity, as shown in Figure 8b. Lacey index denotes a measure of the degree of mixing and it is defined as the following equation [131]:

(19) $$Lacey\;index=\frac{S_{0}^{2}-S^{2}}{S_{0}^{2}-S_{r}^{2}}$$

where $S_{0}^{2}$, $S_{r}^{2}$ and $S^{2}$ are the standard deviation of the unmixed state, the standard deviation of the randomly mixed state and the standard deviation of the sample, respectively. Both the RSD and Lacey index for each particle size show similar results, except for the largest particle size (i.e., 3.0 mm). It has been found that the achievement of slow blending homogeneity in the largest particles is due to differences in blending dynamics. Based on these results, it can be concluded that adjusting the particle size above the critical ratio to ensure similar blending dynamics is desirable for achieving a reasonable computational time in simulations. In addition to these studies, several studies have been conducted to achieve efficient DEM simulation by scaling-up particle sizes [16,106,107,132]. 

##### Young’s Modulus, Shear Modulus and Poisson’s Ratio

Young’s modulus (*E*) denotes the modulus of elasticity for tensile and compressive stresses and the shear modulus (*G*) means the modulus of elasticity for shear stress. They are defined in the following equations [133]:

(20) $$E=\frac{\sigma }{\varepsilon }$$

(21) $$G=\frac{\tau }{\iota }$$

where σ, ε, τ and ι are the stress, strain, shear stress and shear strain. The relationship between Young’s modulus and shear modulus is calculated as follows equation [133]:

(22) E=2(1+v)G

where v is the Poisson’s ratio, which is defined as the ratio between axial strain and shear strain. It is determined the following equation:

(23) $$v=\frac{\Delta d/d_{0}}{\Delta l/l_{0}}$$

where Δ*l/l*_0_ and Δ*d/d*_0_ mean the axial strain and shear strain, respectively. 

**Figure 9:**
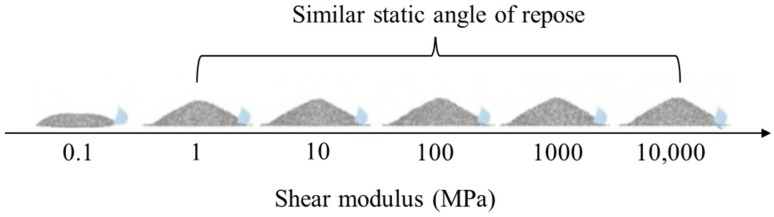
Comparison of the static angle of repose according to the values of shear modulus. The figure is sourced from EDEM^TM^.

In general, shear modulus (or Young’s modulus) can be decreased to reduce the computational time required because they do not significantly affect particle dynamics in simulations of pharmaceutical manufacturing processes [18]. Lommen et al. quantified the effect of shear modulus in three bulk tests (e.g., bulk compression tests, static angle of repose test and penetration test) for shear modulus reduction to achieve a reasonable simulation time [108]. The simulations for the bulk tests were performed using a shear modulus set in the range of 0.01–100,000 MP and the Hertz-Mindlin contact model. The shape of the particles was set to sphere and the values of the other parameters were fixed to evaluate the effect on shear modulus. For the simulations of the bulk tests, the overall particle dynamics was significantly related to shear modulus of low values (0.01–1 MPa), whereas shear modulus of above 100 MPa did not affect the overall particle dynamics. Therefore, these results showed that it is reasonable to reduce shear modulus to accelerate simulation, as long as the particle dynamics is not affected. In addition, a comparison of the static angle of repose according to the value of shear modulus is presented in Figure 9. The results showed that static angle of repose was not significantly changed by shear modulus at a range of 1 to 1000 MPa whereas simulation time was reduced with decreasing shear modulus value. This result supported that it is reasonable to adjust shear modulus to accelerate simulation, as long as the particle dynamics is not affected. 

**Figure 10:**
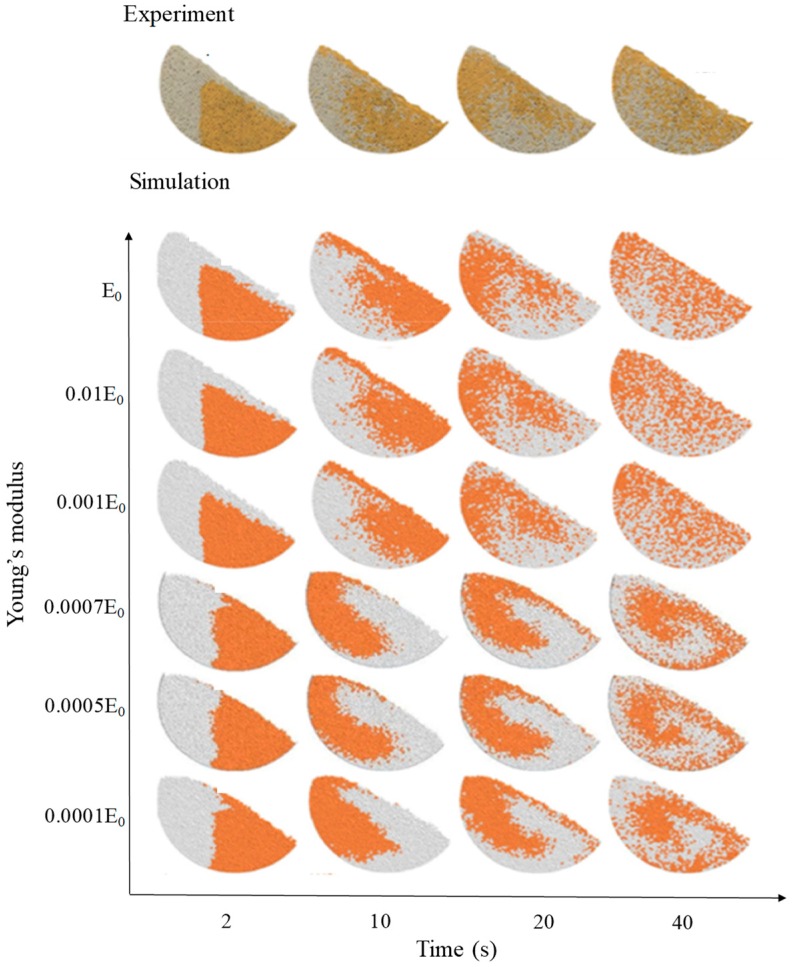
Comparison of the blending dynamics in a rotating drum according to the values of Young’s modulus [109]. The figure was slightly modified with permission from Elsevier, 2017.

In addition, Chen et al. studied the effect of Young’s modulus on particle dynamics in a rotating drum with the Hertz-Mindlin contact model [109]. The values of Young’s modulus were set to 0.0001E_0_, 0.0005E_0_, 0.0007E_0_, 0.001E_0_, 0.01E_0_ and E_0_; E_0_ means the actual Young’s modulus of the particles. The particle size and shape were set to 2 mm and sphere and the other input parameters were fixed. The results showed that the blending dynamic of particles from E_0_ to 0.001E_0_ was similar to each other and were in well agreement with the experimental results, as shown in Figure 10. However, the blending dynamics were different in Young’s modulus from 0.001E_0_ to 0.0007E_0_. On the basis of these results, it can be inferred that adjusting Young’s modulus to the extent that it does not affect the overall particle dynamic is useful for reducing the burden of computational time. 

Besides these moduli of elasticity, Zhou et al. investigated the effect of Poisson’s ratio on particle bulk behavior (i.e., static angle of repose) with modified Hertz-Mindlin contact model [134]. In this study, Poisson’s ratio was set in the range of 0.1–0.7 and the other input parameters were fixed. The static angle of repose measured for these Poisson’s ratio was 26–30°. Therefore, it was confirmed that an increase in Poisson’s ratio showed a slight increase in the static angle of repose but did not have a considerable effect. Moreover, the Poisson’s ratio of the material involving in the pharmaceutical manufacturing processes was generally determined to be within the range of 0.2–0.4 [16,18,25,135,136,137]. Based on these studies, it can be inferred that the shear modulus (or Young’s modulus) and Poisson’s ratio may be defined more flexible than other input parameters.

#### 2.6.2. Interaction Parameters

##### Coefficient of Restitution, Static Friction, Sliding Friction and Rolling Friction

The interaction parameters include coefficient of restitution, coefficient of static friction, coefficient of sliding friction and coefficient of rolling friction. The coefficient of restitution (*e*) is defined as the ratio of the velocity difference before and after the contact and is calculated from the following equation [138,139]:

(24) $$e=\frac{V_{1}^{\prime }-V_{2}^{\prime }}{V_{1}-V_{2}}$$

where, the *V*_1_ and *V*_2_ mean the impact velocity of particle 1 and particle 2 before contact and *V*_1_′ and *V*_2_′ mean the rebound velocity of particle 1 and particle 2 after contact. The coefficient of static friction ($\mu_{st}$) is determined as the ratio of the static friction force (*f_st_*) and normal force (*f_n_*) of material, as shown in the following equation:

(25) $$\mu_{st}=\frac{f_{st}}{f_{n}}$$

The coefficient of sliding friction ($\mu_{sl}$) is defined as the ratio of the sliding friction force (*f_sl_*) and normal force (*f_n_*) of material and the coefficient of rolling friction ($\mu_{r}$) is defined as the ratio of the rolling friction force (*f_r_*) and normal force of material, as shown in the following equations:

(26) $$\mu_{sl}=\frac{f_{sl}}{f_{n}}$$

(27) $$\mu_{r}=\frac{f_{r}}{f_{n}}$$

**Figure 11:**
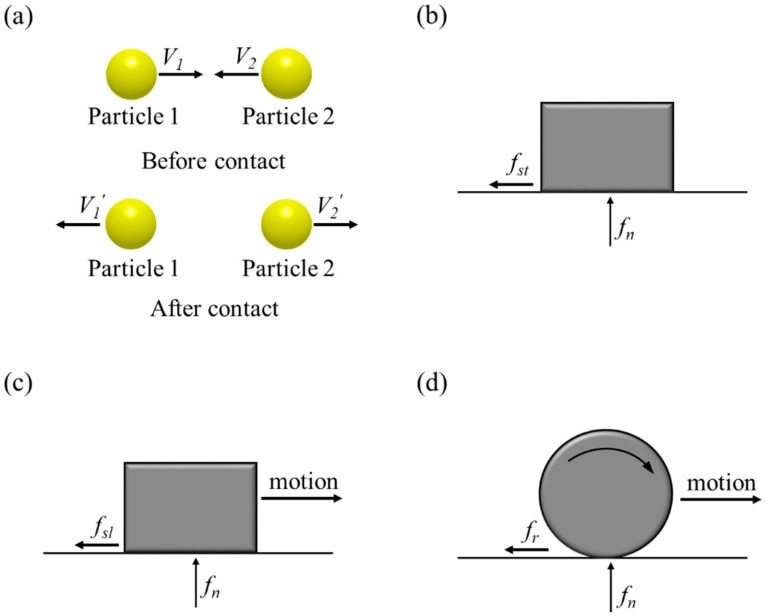
Illustration of the interaction parameters: (**a**) coefficient of restitution, (**b**) coefficient of static friction, (**c**) coefficient of sliding friction and (**d**) coefficient of rolling friction. (*V*_1_ and *V*_2_: impact velocity of particle 1 and particle 2; *V*_1_′ and *V*_2_′: rebound velocity of particle 1 and particle 2; *f_n_*, *f_st_*, *f_sl_* and *f_r_*: normal force, static friction force, sliding friction force and rolling friction force.)

Based on Equations (23)–(26), these interaction parameters can be presented as in Figure 11. 

**Figure 12:**
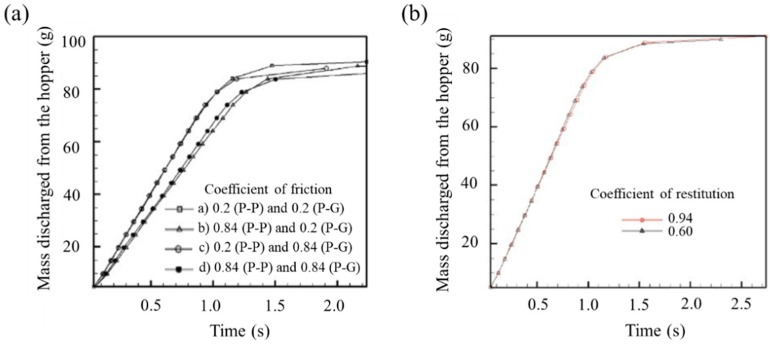
Effect of interaction parameters on hopper discharge profiles: (**a**) coefficient of friction and (**b**) coefficient of restitution [140]. The figures were slightly modified with permission from Elsevier, 2008.

Interaction parameters have a significant effect on pharmaceutical particle dynamics because they are involved in particle-particle (P-P) and particle-geometry (P-G) contact. Anand et al. studied the effects of the coefficient of restitution and friction on hopper discharge dynamics [140]. In this study, the coefficient of friction was divided into four cases as follows: (a) 0.2 (P-P) and 0.2 (P-G); (b) 0.2 (P-P) and 0.84 (P-G); (c) 0.84 (P-P) and 0.2 (P-G); and (d) 0.84 (P-P) and 0. 84 (P-G) and the coefficient of restitution was set to 0.60 and 0.94 with identical values for P-P and P-G, respectively. The hopper discharge profiles according to the coefficient of friction and restitution are presented in Figure 12. In the case of the coefficient of friction, it was observed that the friction between P-P had a much greater effect on hopper discharge rate than P-G. On the contrary, the coefficient of restitution did not have a significant effect on hopper discharge rate. Based on these results, it was inferred that the coefficient of friction plays an important role in particle dynamics, whereas the coefficient of restitution does not. 

**Figure 13:**
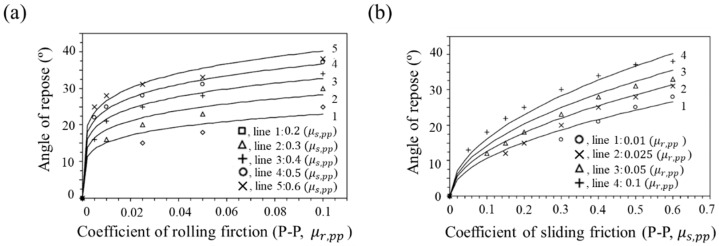
Effect of interaction parameters on static angle of repose: (**a**) coefficient of rolling friction and (**b**) coefficients of sliding friction [134]. The figures were slightly modified with permission from Elsevier, 2002.

In addition, Zhou et al. studied the effect of rolling and sliding friction coefficients on static angle of repose [134]. The relationship between rolling and sliding friction coefficients and static angle of repose is shown in Figure 13. It was observed that the increase in rolling friction coefficient ($\mu_{r,\;pp}$) in the given sliding friction coefficient ($\mu_{s,\;pp}$) resulted in an increase in angle of repose and the increases in sliding friction coefficient ($\mu_{s,\;pp}$) in the given rolling friction coefficient ($\mu_{r,\;pp}$) resulted in an increase in static angle of repose. Therefore, this study showed that rolling and sliding friction coefficients have a significant effect on the static angle of repose of particles.

##### Calibration Method for Input Parameters

Interaction parameters (i.e., coefficient of restitution, coefficient of static, sliding and rolling frictions) should be exactly defined because they are directly involved in particle-particle and particle-geometry contact. However, the interaction parameters for materials in the pharmaceutical industry are not easy to be measured directly [49]. Therefore, a calibration method has been proposed to determine not only interaction parameters but also material properties [112]. Table 1 shows the calibration methods and related DEM input parameters. In general, the calibration method is conducted based on a bulk test of particles. That is, the calibration method is conducted by performing bulk tests of particles and simulating the same test using arbitrary input parameters. Next, the input parameters are repeatedly changed in the simulation until the simulation result is similar to the actual result of the bulk test [112,141]. However, there is a limitation with this method because the input parameters defined through this calibration method may be different from the actual physical meaning of the input parameters [105]. In addition, the input parameters defined by the calibration method with the bulk test may not be accurate because the bulk properties are represented by a combination of two or more parameters [142]. To overcome these problems, one or more experiments can be performed to obtain a single parameter value or two or more experiments should be performed to obtain a set of parameter values [112]. In general, there are no standardized methods for bulk characterization experiments used in calibration methods. However, numerous methods have been studied to define input parameters [142,143,144,145,146]. 

**Figure 14:**
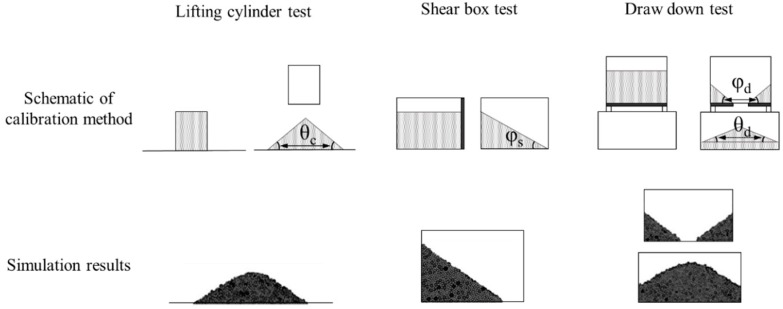
Calibration method based on lifting cylinder test, shear box test and draw down test to determine interaction parameters. (*θ*_c_: static angle of repose for the lifting cylinder test; *φ*_s_: shear angle for the shear box test; *θ*_d_ and *φ*_d_: static angle of repose and shear angle for the draw down test, respectively.) [150]. The figures (i.e., simulation results) were slightly modified with permission from Elsevier, 2019.

Roessler et al. studied a simulation for three bulk tests (i.e., lifting cylinder test, shear box test and draw down test) to determine interaction parameters, as shown in Figure 14 [150]. The lifting cylinder test was performed by filling a cylinder with materials and then lifting the cylinder at a constant velocity to measure the static angle of repose (*θ*) of the pile of materials accumulated below. The shear box test was performed by filling a box with materials and then opening the right flap to allow the materials to escape and the angle of slope formed by the remaining materials was measured. This angle of slope is referred to as shear angle (*φ*). The draw down test was performed with an upper box and a lower box. The upper box has a flap at the bottom center to allow discharge of materials in the lower box. Through discharge of materials from the flap, the remaining materials in the upper box was measured for shear angle and the accumulated pile in the lower box was measured for static angle of repose. The static angle of repose and shear angle measured based on these three bulk tests were compared with those obtained from simulations for the same three tests. The resulting interaction parameters showed similar results to the actual bulk tests. Therefore, this study confirmed that the combination of individual bulk tests can be used to determine accurate interaction parameters. However, measurements of the angle and height of the pile might be highly sensitive to the operator’s judgement. Therefore, a relatively large error between simulations and measurements should be considered. 

**Figure 15:**
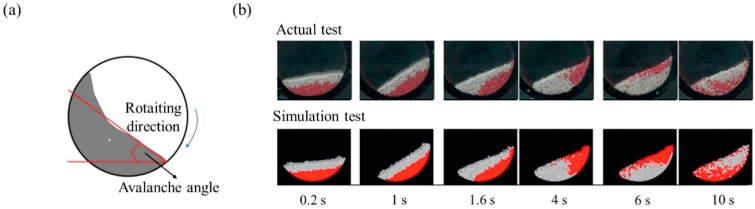
Calibration method based on dynamic angle of repose: (**a**) description of avalanche angle [165] and (**b**) comparison of dynamic angle of repose between the actual test and simulation results [153]. The figures were slightly modified with permission from Elsevier.

The dynamic angle of repose method can present powder flowability and cohesion property. Generally, a drum is half filled with powder and it rotates around its axis with specific angular velocities. The avalanche angle is calculated, which corresponds to the angle where the powder was at the maximum position before the start of the avalanche, as shown in Figure 15a [164,165]. This method needs to be replicated by DEM simulation and both the average avalanche angle and the standard deviations need to be derived and compared with the experimental values. The static part and the frictional part of the powder flow might affect dynamic angle of repose. Therefore, the simulation value might be influenced by DEM parameters, such as friction coefficient, particle shape and cohesive force. Hu et al. conducted faster calibration of the bulk properties of the material, including interaction parameters such as coefficient of friction [153]. In this study, simulation was performed based on the dynamic angle of repose in a rotary drum by adjusting the coefficient of friction. Quantitative comparison between the simulation result and the actual bulk test result over time, as analyzed using the derived coefficient of friction, is shown in Figure 15b. The coefficient of friction obtained by the calibration method is similar to the actual dynamic angle of repose. These results showed the suitability of the calibration method based on the dynamic angle of repose for the accurate prediction of particle dynamics.

**Figure 16:**
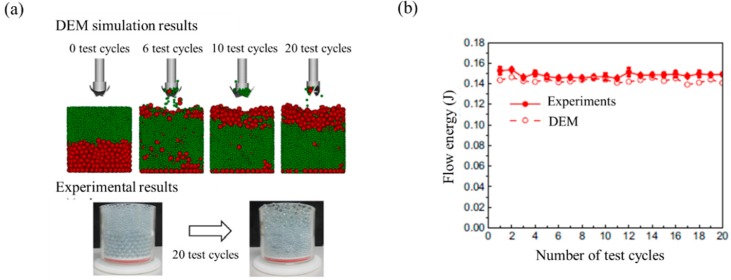
Calibration method based on FT4 rheometer test: (**a**) observation of particle segregation in DEM simulation and experiments and (**b**) comparison of flow energy between DEM simulation and experiments [154]. The figures were slightly modified with permission from Elsevier, 2016.

The FT4 rheometer test can be used to measure a range of powder properties, such as flowability and shear strength. When an impeller blade passes through a powder bed, the force and torque on the impeller blade are detected. The movement of the blade is controlled by a function of vertical velocity, rotational velocity and the helix angle of the blade. The total energy required to mix a powder bed is calculated using the following Equation (27).

(28) $$E_{t}=\overset{t}{\underset{0}{\sum }}v_{v}F\left(t\right)\Delta t+\theta_{r}T\left(t\right)\Delta t$$

where Δt is the data write out interval, $v_{v}$ is the blade vertical velocity, $\theta_{r}$ is the blade rotational velocity and *F*(*t*) and *T*(*t*) are the instantaneous vertical force and torque on the blade at time t, respectively. Yan et al. performed a calibration using an FT4 rheometer to define interaction parameters [154]. In this study, experiments and simulations were performed on an FT4 rheometer and calibration was carried out by mainly adjusting static and rolling friction coefficients. Flow energy was evaluated from the results of the simulation and experiment, as shown in Figure 16. The results of the simulation with the calibrated interaction parameters showed consistent particle segregation and flow energy with those shown in the actual experimental results.

**Figure 17:**
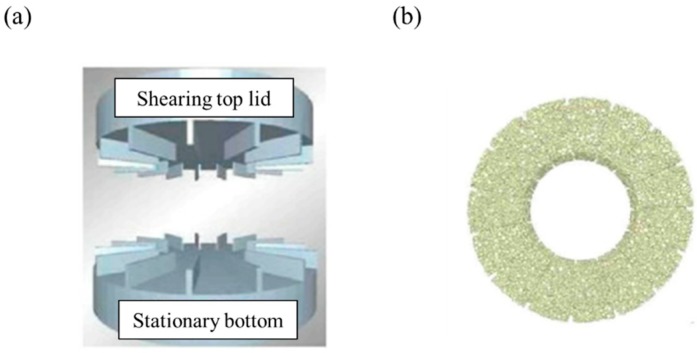
Schematic of a ring shear cell test: (**a**) shear cell tester and (**b**) particle simulated inside the shear cell tester [158]. The figures were slightly modified with permission from AIP Conference Proceedings, 2009.

The ring shear cell test is suitable for blending applications, especially when a powder is pressurized during blending or while kept inside a container, as well as for predicting the conditions under which caking is likely to occur [158]. The instrument is composed of a top lid and a bottom disk, as shown in Figure 17. A powder is contained in an annulus, restricted by co-axial cylindrical walls and is rested on a stationary bottom disk, covered with a top lid. Both the top lid and bottom surface have geometric spaces (teeth) oriented radially and uniformly spaced around the annulus. The top lid rotates with a controlled angular speed and applies a normal load to the powder. This method can present major consolidation stress and unconfined yield strength [166]. During the pre-shear step, the consolidation stress describes the normal and shear stresses in the cell. In addition, the cohesive strength presents the unconfined yield strength during the pre-shear step. The test is then repeated over a range of consolidation states to establish the flow function relationship, flow function coefficient and time flow function relationship of the powder. Flow function relationship is the relationship between consolidation pressure and the cohesive strength (unconfined yield strength) of the powder, whereas time flow function is the relationship between consolidation pressure and the material’s cohesive strength after it has been stored at rest. Simons et al. performed a simulation using a ring shear cell tester to conduct a sensitivity study of input parameters before the calibration [157]. In this study, the effect of input parameters (i.e., Young’s modulus, Poisson’s ratio, coefficient of restitution, static and rolling friction coefficients and particle density) on pre-shear stress was investigated. Young’s modulus and static and rolling friction coefficients were confirmed to have a significant effect on pre-shear stress but Poisson’s ratio and coefficient of restitution did not have a significant effect. Besides the ring shear test, Keppler conducted a sensitivity study to accelerate the calibration based on the standard shear test referred to as Jenike shear cell [160]. The sensitivity study was performed to identify the effect of input parameters (e.g., density, Young’s modulus, Poisson’s ratio, coefficient of friction, bond normal cohesion and bond tangential cohesion) on the particle dynamics (i.e., internal friction angle and cohesion) obtained by Jenike shear cell. As a result, Young’s modulus, Poisson’s ratio, coefficient of friction and bond tangential cohesion had a significant effect on the particle dynamics, while the density and bond normal cohesion were not significant. On the basis of these studies, a calibration method based on the shear cell test can be usefully utilized for DEM simulation. 

**Figure 18:**
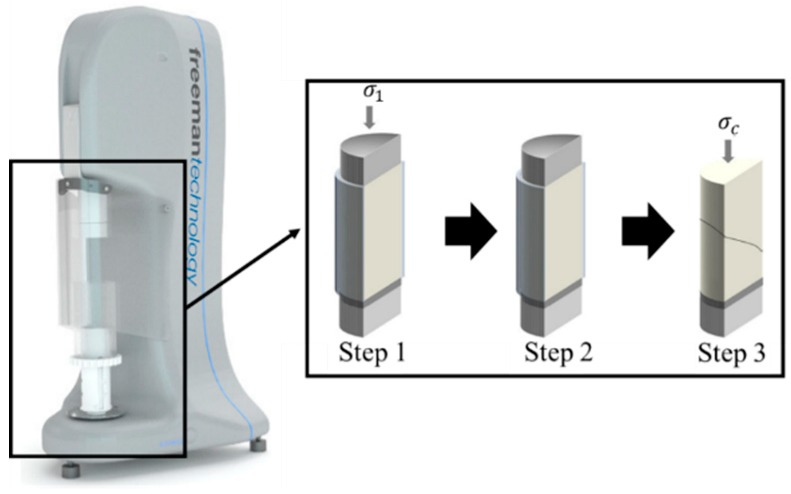
Uniaxial tester of the Freeman Technology (Freeman Technology, Malvern, UK) and schematic representation of the uniaxial test. ($\sigma_{1}$ and $\sigma_{c}$ denote major principal stress and compressive stress, respectively.) The figure is sourced from Freeman Technology.

Uniaxial testers provide information on the flowability of powders and sticky granules in the pharmaceutical industry [167]. As shown in Figure 18, a sample is loaded into a cylinder and consolidated with major principal stress ($\sigma_{1}$) to form a powder column (step 1). Major principal stress and cylinder are removed to afford a free-standing consolidated powder column (step 2). The column is fractured through application of compressive stress ($\sigma_{c}$) and uniaxial unconfined yield strength (step 3). The cohesive powders show a strong bonding between particles with relatively strong inter-particular forces. Moreover, the tensile forces between particles are much weaker in non-cohesive powders than in the cohesive powders. A flow factor can be calculated by dividing the major consolidation strength by the unconfined yield strength. A greater flow factor shows better flowability at any given pressure [168]. This method is related to DEM input parameters, such as static and rolling friction coefficients and contact plasticity ratio. In addition, calibration for defining input parameters based on the uniaxial test has been performed in several studies [161,162,163]. Besides these studies, several other studies have been performed to define interaction parameters based on various calibration methods [105,151,169]. Based on these studies, it can be concluded that this calibration method to define input parameters may have an important role in realizing accurate DEM simulations.

### 2.7. Available DEM Software for the Pharmaceutical Industry 

More recently, the various available DEM software has emerged with the advances in computer hardware. The DEM software can be categorized into two types such as commercial software and open-source software. The former includes EDEM^TM^, Rocky DEM^TM^, Star CCM+, LS-DYNA^®^, PFC 2D (3D) and the latter includes Mercury-DPM, YADE, LIGGGHTS and MFIX-DEM [5,6,170,171]. Based on the development of such available software, a large number of particles can be simulated within a relatively reasonable time [44,110]. Therefore, the application of DEM through the use of such software has been expanding in the pharmaceutical industry. The available DEM software is summarized in Table 2 with the studies related to the manufacturing process in the pharmaceutical industry using each software. 

## 3. Applications of DEM in the Pharmaceutical Manufacturing Process

DEM is useful as a reliable modeling tool for the manufacturing process simulation in the pharmaceutical industry where particle handling processes are prevalent. The application of DEM to the pharmaceutical manufacturing process simulation can enhance the understanding and design of the manufacturing process and accelerate manufacturing process improvement and development [9]. Therefore, the application of DEM through the use of such software has expanded within the pharmaceutical industry. For example, Fu et al. investigated the packing of pharmaceutical powders using DEM simulation [221]. In addition, Mukherjee et al. studied the effect of humidity on pharmaceutical powder flow in simplified hopper [222]. These studies demonstrated that the validity of DEM in the pharmaceutical industry by showing good agreement with DEM simulation and actual experimental results. Moreover, DEM has been applied to manufacturing processes such as tableting, milling, blending, granulation and coating [1,9]. In the tableting process, the die filling simulations were conducted to investigate the parameters (e.g., particle size, fill ratio, particle shape) on the flowability in several studies [223,224]. Also, Garner et al. performed a die compaction simulation using DEM to investigate the microscale behavior of particle during die compaction [209]. The application of DEM to the other manufacturing processes (i.e., milling, blending, granulation and coating) has been extensively studied. In addition, DEM can be used in combination with various modeling tools such as CFD, FEM and population balance model (PBM) for simulation of these processes [15,225,226]. The application of DEM in these manufacturing processes is described in detail in the following section. 

### 3.1. Milling

Milling is a manufacturing process often applied in the pharmaceutical industry to improve the solubility of poorly soluble drugs using equipment such as ball mill, fluid energy mill, conical screen mill, hammer mill and stirred media mill [227,228,229]. Through the milling process, mechanical energy is applied to break down the coarse particles into fine particles such as few micron particles (e.g., typically 2–5 μm) or sub-micron particles (e.g., typically 200–500 nm) [227,230,231]. Lab-scale milling processes allow comparatively rigid control but control of milling when scaled up presents a significant challenge. Therefore, it is necessary to develop a model that can predict the progress of milling in various milling equipment. For this purpose, DEM can be applied to the milling process as a useful modeling tool [179]. Milling simulations are mainly carried out via DEM on the following two aspects—particle fracture and attrition study and particle dynamic study in the milling equipment [4]. The application of DEM to the milling process for various purposes is summarized in Table 3, along with the simulation conditions and predicted results. 

**Figure 19:**
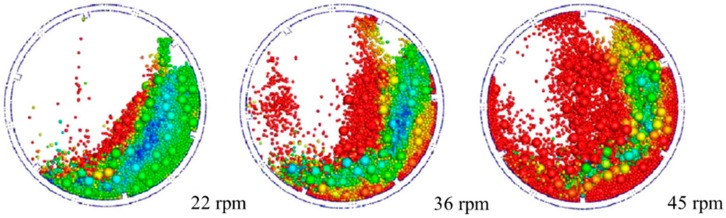
Milling simulation at different rotation speeds [47]. The figure was slightly modified with permission from Elsevier, 2012.

Several studies have been performed to investigate the particle fracture by using DEM in the milling process. Potapov et al. performed a study to investigate the particle fracture induced by mechanical forces using DEM [238]. A detailed investigation of the milling process was conducted by controlling various input parameters (e.g., Poisson’s ratio and Young’s modulus). The results of this study were discussed through dimensional analysis and showed similar results to actual experiments. In conclusion, it has been revealed that the particle size distribution (PSD) induced by the milling process was most affected by alteration of collisional energy and least affected by changing Poisson’s ratio. Wang et al. conducted a study of particle fracture by using DEM of the milling process [47]. This study has investigated the effect of three types of energy inside the milling device on particle fracture through the application of DEM simulation in a ball mill; collision energy, dissipative energy and maximum impact energy. The changes in PSD changes according to the milling time were predicted by the simulation and compared with the actual experimental data and it was shown that the collision energy was directly related to the particle fracture and reliably predicted the PSD changes. The impact of process parameters, such as rotation speed, the loading of the ball and material, were investigated with respect to collision energy in this study, as shown in Figure 19. Several studies investigated the particle dynamics in addition to particle fracture in the milling process. Rajamain et al. performed a study using DEM simulation to describe the charge motion in a tumbling mill [239]. In this study, DEM was applied to investigate the charge motion caused by a ball as a simple physical model with empirical data cannot accurately account for this charge motion. The developed DEM model has been validated through the comparison with experimental data and two-dimensional and three-dimensional DEM algorithms were proposed. As a result, this study demonstrated that DEM was useful for the description of the ball charge motion. Capece et al. conducted a simulation using a combined DEM-PBM approach to simulate the changes of PSD during the ball milling process [233]. In this study, DEM was applied to predict the particle breakage behavior from particle dynamics and PBM was used to predict the changes of PSD based on the particle breakage rate constants obtained from DEM simulations. This study demonstrated the feasibility of the combined DEM-PBM approach to simulate the evolution of PSD in the milling process within a reasonable computational time. Also, a number of studies investigating particle dynamics were performed using various milling equipment (e.g., fluid energy mill, conical screen mill and hammer mill) [180,181,235]. Based on these studies, it can be concluded that DEM serves as a desirable modeling tool for the milling process and helps improve our comprehension of the fracture and dynamics of particles. Several studies have been performed to investigate the particle fracture by using DEM in the milling process. Potapov et al. performed a study to investigate the particle fracture induced by mechanical forces using DEM [238]. A detailed investigation of the milling process was conducted by controlling various input parameters (e.g., Poisson’s ratio and Young’s modulus). The results of this study were discussed through dimensional analysis and showed similar results to actual experiments. In conclusion, it has been revealed that the particle size distribution (PSD) induced by the milling process was most affected by alteration of collisional energy and least affected by changing Poisson’s ratio. Wang et al. conducted a study of particle fracture by using DEM of the milling process [47]. This study has investigated the effect of three types of energy inside the milling device on particle fracture through the application of DEM simulation in a ball mill; collision energy, dissipative energy and maximum impact energy. The changes in PSD changes according to the milling time were predicted by the simulation and compared with the actual experimental data and it was shown that the collision energy was directly related to the particle fracture and reliably predicted the PSD changes. The impact of process parameters, such as rotation speed, the loading of the ball and material, were investigated with respect to collision energy in this study, as shown in Figure 19. Several studies investigated the particle dynamics in addition to particle fracture in the milling process. Rajamain et al. performed a study using DEM simulation to describe the charge motion in a tumbling mill [239]. In this study, DEM was applied to investigate the charge motion caused by a ball as a simple physical model with empirical data cannot accurately account for this charge motion. The developed DEM model has been validated through the comparison with experimental data and two-dimensional and three-dimensional DEM algorithms were proposed. As a result, this study demonstrated that DEM was useful for the description of the ball charge motion. Capece et al. conducted a simulation using a combined DEM-PBM approach to simulate the changes of PSD during the ball milling process [233]. In this study, DEM was applied to predict the particle breakage behavior from particle dynamics and PBM was used to predict the changes of PSD based on the particle breakage rate constants obtained from DEM simulations. This study demonstrated the feasibility of the combined DEM-PBM approach to simulate the evolution of PSD in the milling process within a reasonable computational time. Also, a number of studies investigating particle dynamics were performed using various milling equipment (e.g., fluid energy mill, conical screen mill and hammer mill) [180,181,235]. Based on these several studies, it can be concluded that DEM serves as a desirable modeling tool for the milling process and helps improve our comprehension of the fracture and dynamics of particles.

### 3.2. Blending

Blending is one of the key pharmaceutical manufacturing processes for the preparation of solid dosage forms [240]. Mixtures prepared through the blending process should ensure homogeneity that can directly affect the safety and efficacy of the drug product. Such homogeneity is affected by issues, including agglomeration and segregation, that occur during the blending process. However, it is difficult to detect these issues immediately during the blending process [44]. Furthermore, the blending process should be carefully controlled as active pharmaceutical ingredients (APIs) are present at low concentrations in many dosage forms. Therefore, it is important to have a thorough understanding of the blending mechanism, as well as the material and process parameters [241]. For this reason, the application of DEM to the blending process simulation can be useful [136]. The applications of DEM in blending process simulation in the pharmaceutical industry is summarized in Table 4, with focus on the simulation conditions and predicted results.

**Figure 20:**
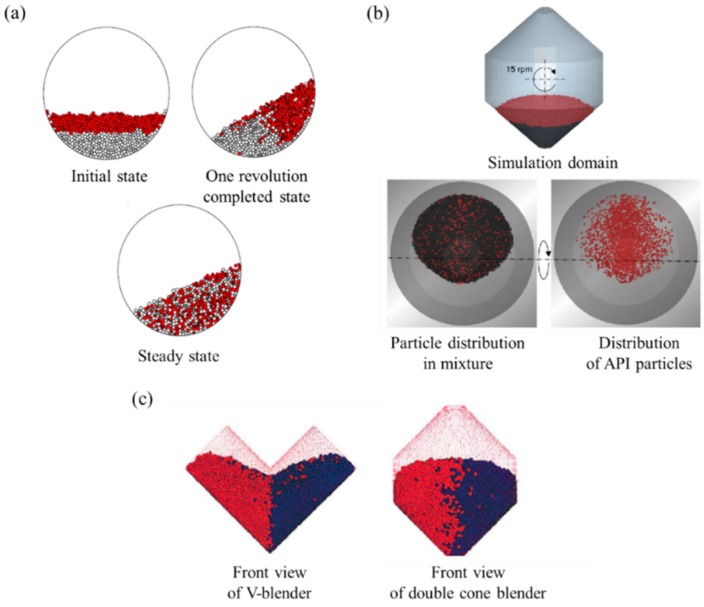
Blending simulation using DEM in (**a**) a cylindrical drum [205], (**b**) a double cone blender [44] and (**c**) V-blender and double-cone blender [185]. The figures were slightly modified with permission from Elsevier.

The application of DEM to the blending process has been discussed in many studies. Specifically, research has focused on how the blending of powders proceeds in rotating devices such as V-blender, double cone blender, bin blender, rotating drum, which are commonly used in the pharmaceutical industry [9,185,186]. Kwapinska et al. conducted a study to investigate the effect of various conditions on the blending process by applying DEM to a powder blending simulation in cylindrical drums, as shown in Figure 20a [205]. These various conditions included not only the particle size but also the process parameters, such as drum diameter, rotational frequency and drum loading. The results of this study confirmed that all conditions affected blending performance in the simulation. Adam et al. performed a blending simulation using DEM in a double cone blender, as shown in Figure 20b [44]. In this study, QbD and DEM were combined to characterize the blending process through the investigation of the effects of material and process parameters on blending quality and blending end points. Moakher et al. performed a comparative study of blending mechanism and blending dynamics by applying DEM was also conducted in two types of blenders—V-blender and double cone blender, as shown in Figure 20c [185]. This study demonstrated that the equipment design of each blender caused a distinct difference in blending dynamics. Through a number of case studies that used DEM in the blending process, it has been confirmed that DEM is a useful modeling tool in a wide range of processes, from manufacturing process efficiency improvement to equipment design, as well as manufacturing process development. 

### 3.3. Granulation

Granulation, specifically wet granulation, is a manufacturing process commonly applied in the pharmaceutical industry for the preparation of solid dosage forms. It is a preferred process as it improves the flowability of fine powders and reduces the possibility of dust generation [251]. In addition, the granulation process can prevent segregation that may occur in the subsequent processes and therefore improves the content uniformity of the final solid dosage forms. In general, wet granulation takes place in two types of equipment—a fluid bed granulator and a high shear granulator. These two types of equipment are technically different from the powder agitation method and the granule growth method. The former spray the binding solution onto the powder kept in the fluidized bed by air flow. Thus, the granules are formed from the attachment of particles to the droplets of the binding solution that contact the fluidized bed. The latter agitates powder by using an impeller and the binding solution is sprayed onto the upper layer of the powder. The droplets of the binding solution are then dispersed in the powder and form granules [252]. Besides these two granulators, the twin screw granulator as the continuous wet granulation equipment has been attracted to the attention of the pharmaceutical industry [253]. The application of DEM to the granulation process performed in such equipment is useful for the prediction of granule characteristics, as well as the provision of insight into the granulation process [254]. In particular, DEM may have a key role in determining the endpoints of the granulation process and scale-up [255]. Examples of the applications of DEM in the granulation process in the pharmaceutical industry are summarized in Table 5, along with the simulation conditions and predicted results.

**Figure 21:**
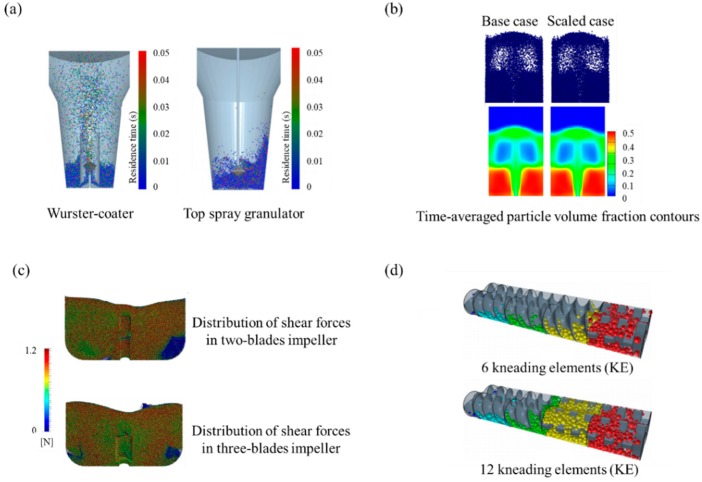
Granulation simulation using DEM in (**a**) fluidized bed granulator of Wurster-coater and top spray [19], (**b**) single-spout fluidized bed granulator [265], (**c**) high shear granulator [17] and (**d**) twin screw granulator [201]. The figures (i.e., (**a**–**c**)) were slightly modified with permission from Elsevier and the figure (**d**) was slightly modified with permission from Springer.

A number of studies have been reported that have coupled DEM with CFD to model fluidized bed granulation processes. In this coupling, CFD and DEM are applied to model the dynamics of fluid and particles, respectively. Fries et al. conducted a study to confirm the fluid and particle dynamics in a fluidized bed granulator during the granulation process [19]. In this study, two types of equipment configuration (e.g., top-spray injection and Wurster-coater) were compared and analyzed in terms of granulation performance by using a DEM-CFD coupling simulation, as shown in Figure 21a. Based on DEM-CFD simulation, the residence time distribution of the particles in the spray zone of each equipment configuration was compared as a result and the effect of the geometry of each equipment on the wetting homogeneity was investigated to understand the performance and specificity of each equipment configuration. Ebrahimi et al. investigated the applicability of Glicksman’s scaling law in the single-spout fluidized bed by using combined DEM-CFD [265]. Based on this scaling law, there was good agreement on the simulation between the base and scale-up cases, as shown in Figure 21b. Therefore, it is suggested that the application of scaling law and DEM-CFD may be promising for single-spout fluidized bed scale-up. In addition, many studies have investigated high shear wet granulation by using DEM. Börner et al. performed a study to investigate the impeller design of a high-shear granulator by using DEM [17]. In this study, the performance of two-bladed and three-bladed impellers was compared. DEM was performed to determine the shear forces applied by the two types of impellers during the granulation process, as shown in Figure 21c. The results of DEM suggested that an impeller with two blades could be an alternative for a robust granulation process, along with other experiments on the performance of the two different types of impellers. In addition to these studies, Barrasso et al. conducted a study using DEM in the wet granulation process in a twin screw granulator composed of various configurations of the screw elements, as shown in Figure 21d [201]. In this study, DEM was combined with PBM, which is a semiempirical model, to predict the attributes of granules (e.g., porosity, particle size distribution and liquid distribution). The predicted results of the developed DEM-PBM model were consistent with the actual experimental results, suggesting that this model can be useful in the designing of a wet granulation process. Based on these studies, it can be concluded that the application of DEM in wet granulation processes is preferred in the process and equipment design, prediction of granule dynamics and scale-up of the granulation process. 

### 3.4. Coating

The tablet, the most common solid dosage form, is often subjected to a coating process to achieve the following objectives—mask the taste of API, add protective functions, control the release of the API and to supplement a second API, often referred to as an active coating. The coating process performed for these various purposes should ensure that the tablet is coated uniformly [266]. In particular, the active coating should be managed as it is directly related to the content uniformity of the tablet [267]. To ensure coating uniformity, coating variability is primarily controlled in terms of inter-tablet coating and intra-tablet coating [26]. However, it is not easy to develop a coating process that ensures coating uniformity. If the coating uniformity is low or highly variable, the coating process must be extended so that all the tablets have a desired coating uniformity, which results in a decrease in process efficiency [18]. To overcome this problem, a DEM simulation that provides insights into the coating process can be applied. The application of DEM to the coating process is summarized in Table 6 with an emphasis on simulation conditions and predicted results. 

**Figure 22:**
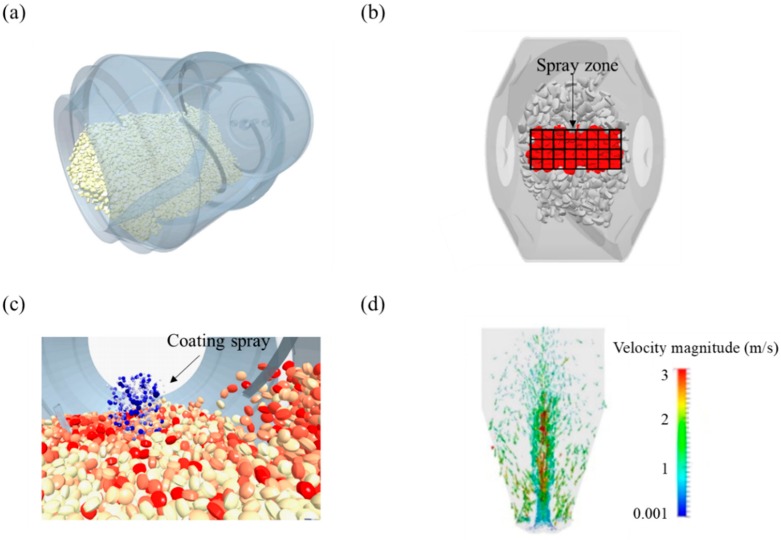
Coating simulation in the coating drum using DEM: (**a**) drum coater geometry [121], (**b**) rectangular spray zone [18], (**c**) coating simulation including the coating spray [191] and (**d**) fluidized bed coating [272]. The figures were slightly modified with permission from Elsevier.

Several studies have reported the application of DEM to model the coating process of tablets. Toschkoff et al. performed a simulation study on the active coating process using DEM simulation in a coating drum, as shown in Figure 22a [121]. In the simulation, the coating process conditions were set to be closest to the actual process. This study was conducted to investigate the effect of process parameters (e.g., rotation speed, filling level, number of spray nozzles and spray rate) on coating uniformity, as well as to achieve a detailed mechanical understanding of coating process. An improvement in coating uniformity was achieved by increasing the number of spray nozzles, increasing the rotation speed and decreasing the filling level. Ketterhagen et al. investigated the effect of various variables on inter-tablet and intra-tablet coating by applying DEM simulation, as shown in Figure 22b [18]. These variables in the simulation included not only process parameters, such as pan speed and pan loading but also tablet shape. In this study, tablet coating variability was predicted through the developed DEM model and validated by using experimental data. Inter-tablet coating variability was shown to be significantly influenced by pan speed and pan loading, whereas the intra-tablet coating variability was considerably affected by tablet shape. Toschkoff et al. developed a model of a spray method in the coating process using DEM simulation, as shown in Figure 22c [191]. In this study, three types of spray methods were implemented and integrated into DEM simulation—spray zone approach, discrete drop method and ray-tracing method. These three different spray methods applied to the same coating process showed similar results. Therefore, this study concluded that the performance of the coating process was highly dependent on the algorithm parameters of the coating process, such as the number of droplets. In addition to these studies, Li et al. conducted a coating simulation study using a DEM-CFD model to determine particle cycle and residence time distribution in a fluidized bed coater [272]. In this study, CFD was applied to simulate fluidization air and DEM was used to simulate the particles involved in the coating process, as shown in Figure 22d. The developed DEM-CFD model showed good predictability in agreement with the experimental results in terms of particle cycle and residence time distribution. Based on several studies using DEM to simulate the coating process, it can be concluded that the application of DEM is useful not only to enhance our understanding of the coating process but also to control coating uniformity, a CQA for tablets.

## 4. Conclusions

Manufacturing process simulation using mechanistic modeling has become increasingly important to overcome the various regulatory and economic problems associated with manufacturing process development in the pharmaceutical industry. Modeling can play a key role in the development of manufacturing processes, including the designing of manufacturing equipment and the enhancement of manufacturing process efficiency. As a tool of mechanistic modeling, DEM is commonly applied in the pharmaceutical industry. DEM is a numerical method that simulates mechanical dynamics, such as velocity, position and motion of individual particles at iterative time steps by solving Newton’s second law and contact models. Contact models play a key role in DEM simulation; they allow the calculation of the contact forces acting among particles. In general, contact models are divided according to particle interactions, such as elasticity and inelasticity. Input parameters (i.e., material properties and interaction parameters) for DEM simulation should be precisely defined for accurate prediction of particle dynamics following the selection of an appropriate contact model. However, the interaction parameters of the pharmaceutical materials are difficult to measure directly. Therefore, various calibration methods have been used to define these parameters. Currently, the computational burden and a relatively long-time required to perform the simulation can limit the application of DEM to the pharmaceutical industry. However, the hurdles might be gradually alleviated by the development of progressive DEM software with the growth of computer hardware. DEM application has various advantages because it not only improves the manufacturing process development but also provides insights into the manufacturing process that are difficult to be obtained from experimentations. Therefore, DEM has been applied widely in various pharmaceutical unit operations, such as milling, blending, granulation and coating. Also, the simulation studies in the pharmaceutical processes have been extended to more complicated modeling such as non-spherical particles, elastic-plastic deformation and non-contact cohesive interaction (e.g., van der Waals force, liquid bridge force and electrostatic force). In the future, DEM application will contribute to the pharmaceutical continuous manufacturing with the goal of real-time release and regulatory perspective for the quality by design approach.

## Figures and Tables

**Table 1:** Frequently used calibration methods and significantly related DEM input parameters.

Calibration Method	Measured Bulk Properties	Related DEM Input Parameters	Ref. Used the Calibration Method
Static angle of repose	Angle of repose	^1^ P-P static friction P-P rolling friction P-P cohesion	[144,147,148,149,150,151]
Dynamic angle of repose	Avalanche angle Dynamic cohesive index	P-P static friction P-P rolling friction JKR surface energy	[151,152,153]
FT4 rheometer	Flow energy P-P friction P-G friction	Bulk density P-P & ^2^ P-G static friction Coefficient of restitution JKR surface energy Surface energy	[154,155,156]
Shear cell test	Bulk density Time flow function Flow function	P-P & P-G static friction P-P & P-G rolling friction Coefficient of restitution JKR surface energy Surface energy	[157,158,159,160]
Uniaxial test	Unconfined yield strength Flow factor	P-P static friction P-P rolling friction Contact plasticity ratio	[161,162,163]

^1^ P-P: interaction between particles. ^2^ P-G: interaction between particle and geometry.

**Table 2:** Summary of the DEM software applied to the process simulation related to pharmaceutical industry.

DEM Software		Company or Developer	Relevant Literature in the Pharmaceutical Industry
Commercial software	EDEM^TM^	DEM solutions Ltd.	[18,19,26,44,63,64,68,121,172,173,174,175,176,177,178,179,180,181,182,183,184,185,186,187,188,189,190,191,192]
	Rocky DEM^TM^	ESSS	[193,194,195,196]
	STAR-CCM+	CD-adapco	[197,198,199,200,201]
	LS-DYNA^®^	LSTC	[202,203,204]
	PFC 2D (3D)	Itasca International Inc.	[135,141,153,205]
Open-source software	MercuryDPM	University of Twente	-
	YADE	SDEC at Grenoble University	[160,206]
	LIGGGHTS	Johannes Kepler University	[207,208,209,210,211,212,213]
	MFIX-DEM	NETL	[214,215,216,217,218,219,220]

**Table 3:** Summary of the examples using DEM for the milling process.

Equipment	Simulation Conditions						Predicted Results Based on the Process Simulation	Ref.
	Contact Model	Simulation Coupling Approach	Simulation Time (s)	Number of Particles	Process Parameters			
Ball mill	Hertz-Mindlin model	DEM-PBM	240	-	∙ Mill-A	∙ Rotational speed: 55 rpm ∙ Grinding ball loading: 35% ∙ Ground material loading: 15%	Particle velocity, power draw, particle flow patterns, collision energy, dissipated energy, maximum impact energy and particle size	[47]
					∙ Mill-B	∙ Rotational speed: 41 rpm ∙ Grinding ball loading: 35% ∙ Ground material loading: 15%		
					∙ Mill-C	∙ Rotational speed: 22 to 67 rpm ∙ Grinding ball loading: 10 to 50% ∙ Ground material loading: 4 to 24%		
		-	-	14,500	∙ Filling level: 20% ∙ Rotational speed: 27.3, 32.7 and 38.2 rpm		Trajectory of particles, collision energy and power draw	[232]
	Hertz-Mindlin no slip model	-	-	-	∙ Milling device: shaker mill	∙ Char ratio (ball to powder mass ratio): 10 ∙ Powder mass: 5 g ∙ Rotational speed: 1054 pm	Energy dissipation rate	[179]
					∙ Milling device: planetary mill	∙ Char ratio: 3 ∙ Powder mass: 30 g ∙ Rotational speed: 300 and 350 pm		
					∙ Milling device: attritor mill	∙ Char ratio: 36 ∙ Powder mass: 50 g ∙ Rotational speed: 200 and 400 pm		
		DEM-PBM	20	Up to 26,320	∙ Rotational speed: 108 rpm ∙ Diameter of grinding medial ∙ Grinding media: 1 kg alumina ∙ Ground material: 100 g silica glass		Impact energy distribution, collision frequency for dissipation energy, specific breakage parameter, material strength parameter and size-independent threshold energy	[64]
		DEM-PBM	10	Up to 14,213	∙ Diameter of grinding media: 14.29 mm ∙ Grinding media: alumina ∙ Ground material: mono-sized silica		Specific breakage rate constant, collision frequency, mass specific energy rate, particle size distribution (PSD)	[233]
		DEM-PBM	20	Up to 24,353	∙ Rotational speed: 108 rpm ∙ Grinding media: 1 kg alumina ∙ Ground material: 100 g silica glass		Collision frequency, specific breakage rate constant, mass specific energy rate, PSD	[234]
Fluid energy mill(Jet mill)	Hertz-Mindlin model	DEM-CFD	0.5	1000	∙ Grind and Feed air pressure: 137.8, 206.8, 275.8, 344.7 and 413.7 kPa ∙ Mass flow rate in feed air inlet: 7.62 × 10^−5^, 1.05 × 10^−4^, 1.31 × 10^−4^, 1.57 × 10^−4^ and 1.83 × 10^−4^ ∙ Mass flow rate in grinding air inlet 1: 7.93 × 10^−5^, 1.07 × 10^−4^, 1.33 × 10^−4^, 1.59 × 10^−4^ and 1.85 × 10^−4^ ∙ Mass flow rate in grinding air inlet 2: 8.18 × 10^−5^, 1.10 × 10^−4^, 1.36 × 10^−4^, 1.62 × 10^−4^ and 1.88 × 10^−4^		PSD, particle and air flow patterns, particle velocity distribution, number of particles in each zone and particle collision frequency and velocity	[180]
Conical scree mill	Hertz-Mindlin model	-	20	5000	∙ Impeller speed: 200, 400 and 1000 rpm ∙ Feed rate: 5000, 10,000 and 20,000 particles per second ∙ Open area: 0.30 and 0.45 ∙ Hole size: 3, 4 and 5 times the diameter of particle size		Collision rate, number of particles in transition zone, average collision numbers and particle number in the conical screen mill	[181]
				10,000				
				20,000				
	Hertz-Mindlin no slip model	DEM-PBM	40	-	∙ Impeller speed: 1750 and 2500 rpm ∙ Feed rate: 4 kg/h ∙ Open area: 25% ∙ Hole size: 990 μm		Particles of different sizes, collision and mass specific energy, material strength parameter, size-independent threshold energy	[182]
Hammer mill	Hysteretic model	-	10	10,000	∙ Impeller speed: 600 and 1140 rpm ∙ Feed rate: 60, 120, 200 g/min ∙ Impeller-wall clearance: 2.5 and 4 mm		Spatial distribution of particles size	[235]
		-	3	4000	∙ Impeller speed: 600 to 1140 rpm ∙ Feed rate: 60 to 110 g/min ∙ Impeller-wall clearance: 2.9 and 3.7 mm		Average particle size, kinetic energy	[236]
Stirred media mill	Hertz-Mindlin model	-	-	-	∙ Diameter of grinding media: 0.8 and 1.2 mm ∙ Grinding media: SiO_2_ and ZrO_2_ ∙ Tip speed of stirrer: 4.0, 6.0 and 9.0 m/s		Cumulative stress energy distribution, spatial distribution of grinding media, number of grinding media contact, powder input, kinetic energy	[210]
		DEM-CFD	-	Up to 119,302	∙ Filling ratio of beads: 0, 50 and 80% ∙ Diameter of beads: 1.0 and 2.0 mm ∙ Stirring rate: 3000, 4500 and 6000 rpm		Fluid velocity, bead and fluid behavior in stirred media mill, bead velocity, average size of aggregated particles, fluid shear power distribution	[237]

**Table 4:** Summary of the examples using DEM for the blending process.

Equipment	Simulation Conditions					Predicted Results Based on the Process Simulation	Ref.
	Contact Model	Simulation Time (s)	Number of Particles	Process Parameters			
V-blender	LSD model	8	11,168	∙ Filling level: 20% ∙ Rotational speed: 15, 30, 45 and 60 rpm		Axial and radial velocities at the cross-sectional plane, particle average speeds, velocity fluctuation, exchange rate between two arms, circulation time in the two arms and dispersion at division and combination steps	[242]
	Modified LSD model	-	420,000	∙ Filling level: 35%	∙ Rotational speed: 15 and 45 rpm	Granular flow and blending dynamics, percentage of particles crossing the axial plane of symmetry, RSD, mean granular velocity and temperature	[243]
			780.000	∙ Filling level: 65%			
	Hertz-Mindlin model	A few seconds	9363	∙ Filling level: 20%	∙ Rotational speed: 15, 30, 45 and 60 rpm	Circulation intensity, particle kinetic energy, particle velocity and axial dispersion coefficient	[183]
			13,108	∙ Filling level: 28%			
			15,917	∙ Filling level: 34%			
			21,534	∙ Filling level: 46%			
	Modified Hertz-Mindlin model	120	225,000	∙ Filling level: 45%	∙ Rotational speed: 30 rpm ∙ Loading profile: top-bottom, front-back and right-left	Granular flow and blending patterns, particle velocity field, torque and degree of mixture homogeneity (RSD)	[244]
			113,200		∙ Rotational speed: 15, 30 rpm ∙ Loading profile: right-left		
	Hertz-Mindlin no slip model and Hertz-Mindlin + JKR model	10	Up to 120,576	∙ Filling level: 30% ∙ Rotational speed: 28, 40 and 60 rpm		Travel distance of particles	[184]
	Hysteretic model	-	15,000	∙ Filling level: 44% ∙ Rotational speed: 15 rpm		Blending mechanism, axial blending flux, particle velocity field and segregation rate	[185]
Double cone blender	LSD model	-	30,000	∙ Filling level: 40% ∙ Rotational speed: 15 rpm		RSD	[44]
	Hysteretic model	10	500,000	∙ Filling level: 40% ∙ Rotational speed: 10, 20 and 30 rpm (horizontal rotation), 0, 10, 20 and 30 rpm (vertical rotation)		Granular flow and blending patterns	[245]
		-	15,000	∙ Filling level: 44% ∙ Rotational speed: 15 rpm		Blending mechanism, axial blending flux, particle velocity field and segregation rate	[185]
Bin (tote) blender	Modified LSD model	-	420,000	∙ Filling level: 35%	∙ Rotational speed: 15 and 45 rpm	Granular flow and blending dynamics, percentage of particles crossing the axial plane of symmetry, RSD and mean granular velocity and temperature	[243]
			780.000	∙ Filling level: 65%			
	Hertz-Mindlin model	502	200,000	∙ Filling level: 40 and 65% ∙ Rotational speed: 6 and 12 rpm		RSD, intensity segregation	[186]
		-	Up to 507, 459	∙ Filling level: 50% ∙ Rotational speed: 10 rpm ∙ Loading profile: left-right and top-bottom		Particle blending patterns, RSD, axial velocity of particles and particle velocity distribution	[246]
		-	261,787	∙ Filling level: 20% ∙ Rotational speed: 45 rpm		RSD, particle blending patterns and particle mean velocity	[247]
			524,580	∙ Filling level: 40% ∙ Rotational speed: 45 rpm ∙ Loading profile: left-right and top-bottom			
			665,980	∙ Filling level: 50% ∙ Rotational speed: 15, 30, 45, 60 rpm ∙ Loading profile: left-right and top-bottom ∙ Inclining angle: 0, 15, 30, 45, 60, 75 and 90°			
			789,610	∙ Filling level: 60% ∙ Rotational speed: 45 rpm ∙ Loading profile: left-right and top-bottom			
			1,015,705	∙ Filling level: 80% ∙ Rotational speed: 45 rpm			
Rotating drum	LSD model	-	Up to 11,860	∙ Filling level: 20 and 30% ∙ Rotational speed: 5.5, 15 and 30 rpm ∙ Drum diameter: 200, 400 and 570 mm		Particle velocity field, number of contacts, mixing time (*t_R_*) and mixing numbers (*N_mix_*)	[205]
		280	278,113	∙ Filling level: 35%	∙ Rotational speed: 11.6 rpm ∙ Particle diameter ratio: 7:3	Active-passive interface, particle trajectory, crossing fraction distribution, particle displacement in the active region and particle residence time in the active and passive region	[248]
			287,660		∙ Rotational speed: 5.6, 7.6, 9.6 and 11.6 rpm ∙ Particle diameter ratio: 6:3		
			300,126		∙ Rotational speed: 11.6 rpm ∙ Particle diameter ratio: 5:3		
			338.677		∙ Rotational speed: 11.6 rpm ∙ Particle diameter ratio: 4:3		
		70	261,946	∙ Filling level: 40%	∙ Rotational speed: 5.6, 7.6 and 9.6 rpm	Axial dispersion coefficient	[249]
			296,939	∙ Filling level: 45%			
	Hertz-Mindlin model	20	Up to 44,296	∙ Rotational speed: 5.5, 15 and 30 rpm ∙ Initial loading profile: side-side and top-bottom		Granular flow and blending patterns and mixing index	[187]
	Hertz-Mindlin + JKR model	300	Up to 10,365	∙ Filling level: 35% ∙ Rotational speed: 25 rpm		Concentration of particles, axial dispersion coefficient and RSD	[25]
	Thornton’s model	Up to 274.26	180	∙ Rotational speed: 20 rpm		Granular flow and blending patterns and velocity field	[250]

**Table 5:** Summary of the examples using DEM for the granulation process.

Equipment	Simulation Conditions						Predicted Results Based on the Process Simulation	Ref.
	Contact Model	Simulation Coupling Approach	Simulation Time (s)	Number of Particles	Process Parameters			
High shear granulator	LSD model	-	5	17,823,551	∙ Blender geometry: 3-blade ∙ Filling level: 70% ∙ Impeller speed: 90 min^−1^		Shear force distribution and kinetic energy	[17]
					∙ Blender geometry: 2-blade ∙ Filling level: 70% ∙ Impeller speed: 30 min^−1^			
		-	3	5000	∙ Impeller speed: 1000 rpm		Particle collision rate, Stoke’s deformation number and consolidation rate constant	[256]
		DEM-CFD	-	-	∙ Impeller speed: 240 rpm		Liquid droplet penetration into a particle bed, droplet impingement on a dynamic particle bed and relative velocity of droplets in vertical direction	[257]
		-	-	8069	∙ Filling level: 13.0%	∙ Impeller speed: 2, 4, 6 and 8 rps	Solid fraction of particles, particle velocity vector and particle velocity	[258]
				16,607	∙ Filling level: 26.8%			
				25,826	∙ Filling level: 41.6%			
				33,354	∙ Filling level: 53.7%			
				41,709	∙ Filling level: 67.2%			
				49,660	∙ Filling level: 80.0%			
	Hertz-Mindlin model	-	10	147,460	∙ Impeller speed: 150, 200, 287 and 345 rpm		Particle velocity field, particle concentration at various regions and number of seeded granules	[188]
		DEM-PBM	-	80,000	∙ Impeller speed: 2 rps		Residence time distribution and volume fractions	[259]
				200,000			Collision frequency	
		-	200	80,000	-		Residence time distribution, volume fraction, particle concentrations from the surface and particle velocity	[260]
	Hertz-Mindlin no slip model	-	44	53,913	∙ Filling level: 25.25 mm ∙ Impeller speed: 443 rpm ∙ Liquid addition rate: 276.77 g/min ∙ Liquid addition time: 44 s ∙ Area flux through spray zone: 2.82 × 10^−3^ m^2^/s		Viscosity of wetted granules, distribution of binder particle and liquid droplets, capillary forces, viscous forces, liquid bridge forces, granules velocity, collision frequency and number of liquid bridges	[261]
	Liquid bridge model	-	10	2132	∙ Impeller speed: 100, 250 and 500 rpm		Total number of liquid bridges	[255]
	Rolling friction model	-	-	8.349	∙ Vessel volume: 1.0 L	∙ Filling level: 50% ∙ Impeller speed: 5,10, 15 and 20 s^−1^	Particle configuration depending on its position, particle velocity filed and particle collision energy	[262]
				28.178	∙ Vessel volume: 3.4 L			
				66,792	∙ Vessel volume: 8.1 L			
				130,454	∙ Vessel volume: 16 L			
Fluid bed granulator	Hertz-Mindlin model	DEM-CFD	15	165,000	∙ Granulator configuration: top-spray ∙ Minimum fluidization velocity: 0.56 m/s		Mean particle residence time, re-circulation time, total particle passes, mean solid volume fraction, mean crossing length, mean particle velocity and particle wetting	[175]
		DEM-CFD	4	150,000	∙ Granulator configuration: top-spray, Wurster-coater and spouted-bed ∙ Fluidization air flow rate: 360 kg/h ∙ Atomizer flow rate: 5.7 kg/h ∙ Gap distance below Wurster: 30 mm		Particle velocity, time-averaged gas velocity and solid volume fraction, particle collision velocity, density distribution and angular velocity	[63]
	Hertz-Mindlin no slip model	DEM-CFD	5	45,000	∙ Granulator configuration: Wurster-coater and top spray granulator ∙ Gas injection velocity: 160 m/s ∙ Fluidization velocity bottom spray: 11 (zone 1), 5 (zone 2) and 5.5 m/s (zone 3) ∙ Fluidization velocity top-spray: 5.5 m/s ∙ Fluidization air flow rate: 600 m^3^/h ∙ Atomizer air flow rate: 7 m^3^/h		Particle position and velocity distribution, Residence time distribution and solid volume fraction, particle collision and collision velocity and mean contact time	[19]
		PBM-DEM-CFD	10	40,000	∙ Inlet volumetric air flow rates: 80, 110 and 130 m^3^/h ∙ Inlet air temperature: 303 and 323 K ∙ Superficial gas velocity: 1.3, 1.9 and 2.2 m/s		Air flow rate, solid volume fraction, particle velocities, compartmental distribution of particles, inter-compartmental particle transfer, particle collision frequencies, particle collision energy, particle residence time in the spray zone and particle temperature	[199]
	Hertz-Mindlin + JKR model	-	0.525	50,000	∙ Atmospheric air temperature: 313 K ∙ Fluidization gas flow rate: 57.1 mm/s		Number of granules, number of bonds and active sprayed particles, adhesive bond energy, granule size distribution and fractal dimension	[263]
Twin screw granulator	Hertz-Mindlin model	DEM-PBM	30	-	∙ Liquid to solid ratio: 0.25 ∙ Screw configuration: feed screw elements and mixing elements ∙ Screw speed: 240 rpm		Number contacts, impact frequency and average particle velocity	[200]
		DEM-PBM	10	1000	∙ Geometrics configurations: various combinations of conveying elements and kneading elements in a total of 4 compartment		Residence time information, particle collision and velocity data	[201]
	Modified Hertz-Mindlin model	-	-	195,916	∙ Filling level: 60% ∙ Screw configuration: short pitch feed screw ∙ Screw speed: 10 rpm		Granular flow, surface velocity vectors, resultant velocity	[264]

**Table 6:** Summary of the examples using DEM for the coating process.

Equipment	Simulation Conditions						Predicted Results Based on the Process Simulation	Ref.
	Contact Model	Simulation Coupling Approach	Simulation Time (s)	Number of Particles	Process Parameters			
Pan coater	Hertz-Mindlin model	-	600	40,000	∙ Filling level: 67%	∙ Rotational speed: 4, 6 and 8 rpm ∙ Spray pattern: a full spray, a band spray with a band parallel to the axis of rotation, five elliptical spray patterns simulating and realistic spray from five spray guns	RSD of concentration, RSD of residence time and residence time distribution	[62]
				60,000	∙ Filling level: 100%			
		-	60	1000	∙ Rotational speed: 20 rpm		Tablet coating thickness and cap-to-band ratios	[211]
	Hertz-Mindlin no slip model	-	60	Up to 1539	∙ Particle loading: 0.7 and 1.0 kg ∙ Rotational speed: 16 to 28 rpm		Tablet orientation in the spray zone appearance frequency, mean circulation time between appearances, mean residence time per pass, inter-tablet coating uniformity and intra-tablet coating uniformity	[18]
		-	1800	Up to 770	∙ Particle loading: 1 kg ∙ Rotational speed: 22 rpm		Intra-tablet coating variability and coating thickness distribution	[177]
		-	120	Up to 1168	∙ Particle loading: 1.5 kg ∙ Rotational speed: 22 rpm		Intra-tablet coating variability and relative asymptotic coating thickness	[178]
	LSD model and hysteretic model	-	60 or 120	-	∙ Filling level: 11.6, 13.5, 18.7 and 24.9% ∙ Rotational speed: 8. 12, 16, 24, 28 and 32 rpm		Average and deviation of residence time, fractional residence time and the dimensionless appearance frequency	[268]
	Hysteretic model	-	12	Up to 90,000	∙ Tilt of the pan: 0, 16 and 32° ∙ Rotational speed: 10, 20 and 30 rpm		Coating variability and frequency distribution of residence time	[46]
	Modified Thornton’s model and hysteretic model	-	6 or 8	4700	∙ Filling level: 10%	∙ Rotational speed: 6, 9 and 12 rpm	Dynamic angle of repose, average cascading velocity and average surface velocity	[269]
				6000	∙ Filling level: 14%			
				7500	∙ Filling level: 17%			
Drum coater	LSD model	-	90	815,602	∙ Particle loading: 230 kg	∙ Rotational speed: 8, 9 and 10 rpm ∙ Number of nozzles: 4, 6 and 8 ∙ Spray rate: 160, 240 and 360 g/min	Inter-tablet coating uniformity, velocity distribution in the spray zone, spray residence time and normalized bed cycle time	[267]
				1,028,368	∙ Particle loading: 290 kg			
		-	36	-	∙ Particle loading: 3, 4, 16.11 and 21.48 kg ∙ Rotational speed: 5, 10.3, 15,4, 20.7 and 25.4 rpm ∙ Circumferential velocity: 8.25, 16.99, 25.4, 34.14, 41.89 cm/s		Tablet velocity, spray residence time and tablet bed residence	[270]
		-	60	26,362	∙ Particle loading: 15 kg	∙ Rotational speed: 10 rpm	RSD of binary mixture, residence time, tablet velocity field, surface velocity of tablet bed and tablet angular velocity	[26]
				31,634	∙ Particle loading: 18 kg			
				36,906	∙ Particle loading: 21 kg			
	Modified LSD model	-	-	10,638	∙ Particle loading: 3 kg (Lab-scale)		Tablet velocity	[171]
				14,184	∙ Particle loading: 4 kg (Lab-scale)			
				57,128	∙ Particle loading: 16.11 kg (Pilot-scale)			
				76,170	∙ Particle loading: 21.48 kg (Pilot-scale)			
	Hertz-Mindlin model	-	25	4200	∙ Rotational speed: 300 rpm		Particle radial and tangential velocity distribution and number of contact	[189]
		DEM-PBM	1000	2263	∙ Filling level: 25%	∙ Rotational speed: 10 and 17 rpm	Inter-tablet coating variability and residence time distributions	[190]
				2694	∙ Filling level: 30%			
	-	-	18	12,446	∙ Coating method: spray zone approach, discrete drop method and ray-tracing method		RSD and coating mass distribution	[191]
		-	90	Up to 14,177	∙ Particle loading: 3, 3.5 and 4 kg ∙ Rotational speed: 16, 18 and 20 rpm ∙ Spray rate: 8, 12 and 16 g/min ∙ Number of nozzles: 2 and 4		Coefficient of variation of the coating mass	[121]
Fluidized bed coater	LSD model	-	20	2400	∙ Jet velocity: 42, 46.2 and 50.4 m/s ∙ Gas velocity: 2.8, 3.08 and 3.36 m/s		Bed behavior, average particle height, bed height, gas pressure drop fluctuations and wet coefficient of restitution	[214]
		DEM-CFD	10	7000	∙ Gas inflow rate: 10, 12.5, 15 and 17.5 m/s ∙ Spacing between the Wurster insert and the solid based of the bed: 1.0, 1.5, 2.5 and 3.0 cm ∙ Slope of the base of the bed: 10, 20 and 30°		Probability distribution functions for the coating volume and inter-tablet coating uniformity	[271]
	Modified LSD model	DEM-CFD	30	32,400	∙ Liquid flow rate: 10−3 m^3^/h ∙ Fluidized gas flow rate: 80.3 m^3^/h ∙ Atomized gas flow rate: 4.32 m^3^/h		Cycle time distribution, residence time distribution and collision velocity	[212]
	Hertz-Mindlin model	DEM-CFD-CVD ^1^	7	15,000	∙ Inlet gas velocity: 5.0, 8.0 and 11.0 m/s ∙ Wall temperature: 1280, 1450 and 1680 K		Layer thickness, deposition rate, fluid dynamic pressure, fluid volume fraction and particle velocity field	[192]

^1^ chemical vapor deposition (CVD).
